# The association between perceived household educational support and HIV risk in young women in a rural South African community (HPTN 068): A cross sectional study

**DOI:** 10.1371/journal.pone.0210632

**Published:** 2019-01-17

**Authors:** Jessica Price, Audrey Pettifor, Amanda Selin, Ryan G. Wagner, Catherine MacPhail, Yaw Agyei, F. Xavier Gómez-Olivé, Kathleen Kahn

**Affiliations:** 1 School of Public Health and Family Medicine, Faculty of Health Sciences, University of Cape Town, Cape Town, South Africa; 2 Nuffield Department of Primary Care Health Sciences, Medical Sciences Division, University of Oxford, Oxford, United Kingdom; 3 Department of Epidemiology, University of North Carolina Gillings School of Global Public Health, Chapel Hill, North Carolina, United States of America; 4 MRC/Wits Rural Public Health and Health Transitions Research Unit (Agincourt), School of Public Health, Faculty of Health Sciences, University of the Witwatersrand, Johannesburg, South Africa; 5 Carolina Population Center, University of North Carolina at Chapel Hill, Chapel Hill, North Carolina, United States of America; 6 Wits Reproductive Health and HIV Institute (WRHI), School of Clinical Medicine, Faculty of Health Sciences, University of the Witwatersrand, Johannesburg, South Africa; 7 Umeå Centre for Global Health Research, Umeå University, Umeå, Sweden; 8 School of Health and Society, University of Wollongong, Wollongong, New South Wales, Australia; 9 Johns Hopkins University, School of Medicine, Department of Pathology, HPTN Laboratory Center, Baltimore, Maryland, United States of America; Sefako Makgatho Health Sciences University, SOUTH AFRICA

## Abstract

**Objective:**

To characterise perceived household support for female education and the associations between educational support and HIV prevalence, HSV-2 prevalence and sexual risk behaviours.

**Methods:**

This cross-sectional study used baseline survey data from the *Swa Koteka HPTN 068* trial undertaken in Mpumalanga, South Africa. The study included 2533 young women aged 13–20, in grades 8–11 at baseline. HIV and HSV-2 status were determined at baseline. Information about patterns of sexual behaviour and household support for education was collected during the baseline survey. Linear regression and binary logistic regression were used to determine associations between household support for education and both biological and behavioural outcomes.

**Results:**

High levels of educational support were reported across all measures. HIV prevalence was 3.2% and HSV-2 prevalence was 4.7%, both increasing significantly with age. Over a quarter (26.6%) of young women reported vaginal sex, with 60% reporting condom use at last sex. The median age of sexual debut was 16 years. Household educational support was not significantly associated with HIV or HSV-2; however, the odds of having had vaginal sex were significantly lower in those who reported greater homework supervision (OR 0.82, 95%CI: 0.72–0.94), those who engaged in regular discussion of school marks with a caregiver (OR 0.82, 95%CI: 0.71–0.95) and when caregivers had greater educational goals for the young woman (OR 0.82, 95%CI: 0.71–0.96). In contrast, greater caregiver disappointment at dropout was significantly associated with reported vaginal sex (OR 1.29, 95%CI: 1.14–1.46).

**Conclusion:**

Young women in rural South Africa report experiencing high levels of household educational support. This study suggests that greater household educational support is associated with lower odds of having vaginal sex and engaging in risky sexual behaviour, though not with HIV or HSV-2 prevalence.

## Introduction and background

South Africa continues to have the highest rates of HIV worldwide. There are an estimated 6.4 million people living with HIV in the country (12.2% prevalence), with 469 000 new infections each year in those over 2 years old. Young women aged 15–24 years remain the highest risk group for new infections. The incidence in this group is estimated to 2.5%; more than 4 times greater than the incidence in men of the same age[1].

In trying to understand the additional risk experienced by young women, research has highlighted the importance of formal education. School attendance and better education in females have been shown to improve a variety of health outcomes, including reducing the risk of HIV, teenage pregnancy and sexual risk behaviours [2–8]. However, a lack of education alone does not explain the substantially higher risk of HIV acquisition of young women. Increasingly, research points to the role of the parent-child relationship and to the protective effects of greater parental monitoring [9]. “Parental monitoring” generally refers to parents’ knowledge of their child’s whereabouts and activities [10–14]. Various parental monitoring scales are used to assess whether parents are aware of their child’s whereabouts after school, who their friends are, and the enforcement of curfews [10–14]. Parental monitoring appears to reduce sexual risk behaviour amongst male and female adolescents via two mechanisms: firstly by reducing involvement in rebellious/deviant peer groups, and secondly by reducing the opportunities for sexual intercourse simply by limiting unsupervised time [9,10,15–18].

However, the parent-child relationship is more nuanced and complex than can be captured by parental monitoring alone. Brook et al. refer to the “positive parent-child relationship” which mediates the effects of environmental factors by moulding personal characteristics of the child, including self-esteem and behavioural attributes [19]. Increasingly, research has attempted to evaluate the role of other dimensions of the parent-child relationship, suggesting that low levels of parent-child communication, low perceived parental trust and neglectful or authoritarian parenting styles act as independent risk factors for risky sexual behaviour [20]. Negotiated unsupervised time has been shown to increase sexual activity, though it has been argued that it breeds responsibility and encourages a healthier exploration of sexuality, with greater condom use [20].

There is limited work that has attempted to examine the parent-child relationship in so far as it relates to support for education. Where it does exist, it is limited to the question of parental supervision of homework or attendance of school functions (where greater parental involvement has been shown to improve academic performance of the child) [21–24]. Furthermore, as highlighted by van Wyk and Lemmer [25], little attention has been paid to the question of context, and whether caregivers’ involvement with schooling is different in non-nuclear families. Non-nuclear families are a common reality in southern Africa where the HIV/AIDS epidemic has resulted in many single and double orphaned youth[26]. Additionally, no research has attempted to bridge the gap between greater household support for education and a possible effect on adolescent sexual risk behaviours and HIV risk; a significant gap in the literature given the ever-growing body of evidence tying greater education to reduced HIV risk [2–8]. This study addresses such questions directly and aims to characterise the patterns of household educational support, and to consider the variable effects of different elements of support on HIV and Herpes Simplex Virus 2 (HSV-2) risk, as well as sexual risk behaviours such as condom use and age of sexual debut in young women. The underlying hypothesis is that increased household interest in education would improve adolescent school attendance and educational attainment which, in turn, is associated with lower HIV risk. In addition, household interest in education, as part of a positive caregiver-child relationship, cultivates greater self-esteem, enabling young women to be more assertive in negotiating sexual relationships and condom use.

## Materials and methods

### Study design and setting

This cross-sectional study involved analysis of baseline data collected as part of the *Swa Koteka* Cash Transfer Trial (HPTN 068) [27]. Briefly, the study was located in Agincourt, Bushbuckridge–a rural area in Mpumalanga, South Africa. This region remains relatively underdeveloped with high levels of poverty. Electricity, whilst available, is too expensive for many households and there is often no piped water to houses. Among the 28 villages in which this study was conducted, each village has a primary school and most have a high school, though infrastructure is poor and education standards remain low. Access to healthcare is via two health centres and six clinics. There are three district hospitals 25-60km away. Social grants, including child grants and pensions are a significant source of household income [28]. The study was nested in the Agincourt Health and Socio-Demographic Surveillance System (HDSS) site in the area, which provided a sampling framework for the trial [28].

### Eligibility criteria

The *HPTN 068 study* included 2533 young women aged 13–20 years old and in grades 8–11 at enrollment. Where there was more than one eligible young woman in the household, the “next birthday” method was used to select which individual would participate. The “next birthday” method allows for random selection by choosing the person (from all eligible participants) who will next have their birthday for recruitment into the study. Young women who were married or pregnant by self-report at the time of enrollment were not eligible[29].

### Data collection

All households within the Agincourt HDSS site known to have young women eligible for participation were contacted by trained field workers. A baseline household survey was conducted with a household representative between March 2011 and December 2012. The enrolled young woman completed her own baseline behavioural survey at the study offices. HIV and HSV-2 status of the young women were determined at baseline, though were not used as exclusion criteria for participation in the study [27].

### Measures

This study considered both biological and behavioural outcomes collected at baseline. The biological outcomes of interest were HIV and HSV-2 status. Young women were screened for HIV infection using two HIV rapid tests performed in parallel (the Determine HIV-1/2 test [Alere Medical Co, Matsudo-shi, Chiba, Japan] and the Uni-gold Recombigen HIV test [Trinity Biotech, Bray, County Wicklow, Ireland]). If a reactive result was obtained for one or both tests, a confirmatory test was performed using the GS HIV-1 Western Blot assay (Bio-Rad Laboratories Inc. Redmond, WA, USA). If the Western blot was positive or indeterminate, a second Western blot test was performed using a new sample collected within 2 weeks of the initial test. Young women were diagnosed as HIV infected if both Western blot tests were positive [29]. Baseline HIV status was confirmed at the HPTN Laboratory Center.

HSV-2 testing was performed at study sites using the Kalon assay (Herpes Simplex Type 2 IgG ELISA, Kalon Biologics, Ltd., Guildford, UK) with a cut-off of 1.5. This test has been validated in African populations [30]. HSV-2 results were confirmed by repeating the Kalon assay at the HPTN Laboratory Center with the same sample [29].

Behavioural outcomes, including engaging in vaginal sex, condom use during last sexual intercourse and age of vaginal sexual debut were assessed by self-report. Most questions were answered using Audio Computer-Assisted Self-Interview (ACASI) software [29]. ACASI is computer-based technology that allows participants to complete a survey with sensitive questions on a computer with headphones that reads questions out loud. Multiple studies have shown that the use of this technology increases accurate reporting of sensitive behaviours, including sexual activity [31,32].

The exposure of interest in this study, namely household interest in education, was assessed using six questions (Table 1). These questions were adapted from the National Education Longitudinal Study (NELS) in 1988, which in turn have formed the basis of multiple studies assessing the association between parental involvement in adolescent education and student achievement [33–35], and which were identified as common metrics for parental involvement in education in a meta-analysis by Fan and Chen in 2001 [36]. Questions were adapted to be appropriate to the rural South African setting.

**Table 1:** Household interest in education (baseline questionnaire 2011–2012).

Question		Answer Scale
1.	How often does []* check to see that you’ve done your school work?	Never/ Sometimes/ Always
2.	How often does []* help you with your school work?	Never/ Sometimes/ Always
3.	During the 2010 school year, how often did you discuss things you studied in class with []*?	Never/ Sometimes/ Always/ N/A (not in school)
4.	During the 2010 school year, how often did you discuss your marks with []*?	Never/ Sometimes/ Always/ N/A (not in school)
5.	How far in school does []* want you to go?	Finish the current year and no more/ Pass matric/ University or technikon
6.	If you dropped out of school, how disappointed would []* be? If you have previously dropped out of school, tell us how disappointed they were at that time.	Not at all/ Somewhat/ Very much

* [] was replaced by the person identified as most involved in the young woman’s education.

The authors chose to present each of the components of support for education independently to allow the variable effect of each measure to be evaluated, rather than creating a composite indicator of overall household educational support. Questions used 2010 as the reference year indicating the most recently completed academic year for young women enrolled into the study in 2011. For those enrolled in 2012, the questions were updated to read “2011”. Each young woman was also asked to identify the individual most involved in their schooling (mother/father/older brother/older sister/aunt/uncle/grandparent/ cousin/other adult who is not a blood relative).

Parental education was included due to its likely effect on socioeconomic status and its potential confounding effect on involvement in the child’s education. During the household survey, information about household membership, household food and non-food consumption, government grant receipt, health status of household members, education and employment status of household members among other topics was collected. All questionnaires were translated into Shangaan (the first language of the majority of households in the area) and back translated into English to ensure the meaning of the questions remained.

### Analysis

The outcome variables HIV and HSV-2 status were each coded as 1 if positive and 0 if negative. Vaginal sex was coded as 1 if the participant reported ever having had vaginal sex and 0 if she had not. Condom use at last sex was coded as 1 if the participant reported having used a condom when they last had vaginal sex, and 0 if she did not use a condom when she last had sexual intercourse. Age of sexual debut was reported in years as the age at which the young woman first had vaginal sex.

Exposure variables were coded 1/2, 3 where 1 = “never”, 2 = “sometimes”, 3 = “always” referring to frequency of homework supervision, help with homework, discussion of studies and discussion of school marks. Perceived educational goals was coded as 1 = “Finish the current year and no more”, 2 = “Pass matric”, 3 = “University or technikon”, while disappointment at dropout was coded as 1 = “not at all”, 2 = “somewhat” and 3 = “very much”.

Binary logistic regression was used to model the association between the exposures of interest and each of the biological outcomes and behavioural outcomes (including HIV, HSV-2, vaginal sex and condom use at last sex). Binary logistic regression was chosen over other regression models such as probit, as coefficients can be interpreted in terms of odds ratios. Linear regression was used to model the association between exposures and age of sexual debut, which was analysed as a continuous variable. All models examined the independent association between exposures and each of the outcomes of interest, whilst identifying and removing confounding. The final models were only adjusted for age and grade at enrollment as other potential confounders (including who was identified as the primary caregiver, and the level of parental education) did not result in a change in the coefficient of the exposures of interest. All analysis was conducted using Stata12.

### Ethical considerations

This analysis was approved by University of Cape Town Human Research Ethics Committee. The *HPTN 068* trial was approved by the University of Witwatersrand Human Research Ethics Committee, the Mpumalanga Province Health Research and Ethics Committee, and by the ethics committee at the University of North Carolina. Of note, the majority of participants enrolled at baseline were below the legal age of consent (18 years old). In such cases, the young woman provided written assent, whilst the parent/ guardian was required to provide written consent for enrollment.

## Results

This study included 2533 black rural South African young women. The mean age of participants was 15 years, with participants fairly evenly divided amongst grades 8–11 at enrollment (20–27% per grade as shown in Table 2). Mothers were identified as the primary caregiver in 65% of cases, followed by grandparents in 14% of cases and fathers in 8% of cases. Other individuals (siblings, aunts/uncles, cousins and non-blood relatives) were identified as the primary caregiver in less than 12% of cases. The majority of parents had some degree of formal education (Table 2), with 23% of mothers having started or completed primary school and 48% having started or completed secondary school. Similar rates were reported for fathers with 19% having started or completed primary school and 42% who had started or completed secondary school.

**Table 2:** Demographic characteristics of participants.

Variable	Participants (n = 2533)	
	n	Percent*
Mean age in years ± sd	15.52 ± 1.66	-
Grade at enrollment		
Grade 8	640	25
Grade 9	682	27
Grade 10	699	28
Grade 11	512	20
Primary caregiver		
Mother	1664	66
Grandparents	359	14
Father	206	8
Older sibling	169	7
Aunt/uncle	108	4
Cousins	3	0.1
Other adult-not blood relatives	24	1
Orphan		
Non-orphan (both biological parents alive)	1723	71
Single orphan	575	24
Double orphan	117	5
Maternal education		
No education	424	17
Some/completed primary school	576	23
Some/completed secondary school	1233	49
Tertiary education (technikon/university)	88	3
Unknown	212	8
Paternal education		
No education	432	17
Some/completed primary school	475	19
Some/completed secondary school	1058	42
Tertiary education (technikon/university)	102	4
Unknown	466	18

*percents may not add to 100 due to rounding

### Outcomes

HIV prevalence at baseline was 3.2%. HIV prevalence increased significantly with age (from 2.2% prevalence in those aged 13–14 years to 10.2% prevalence in the oldest age group 19–20 years, p<0.001) (Table 3). This relation to age is similarly present in the case of HSV-2 status with 4.7% prevalence overall, increasing from 0.65% prevalence in the youngest age group to 18.8% in the oldest (p<0.001) (Table 3).

**Table 3:** Outcomes overall and by age group.

Outcome	Total (%)	Age 13–14 (%)	Age 15–16 (%)	Age 17–18 (%)	Age 19–20 (%)
	n = 2533 (100)	n = 773 (31)	n = 1075 (42)	n = 557 (22)	n = 128 (5)
HIV Positive	81 (3)	17 (2)	25 (2)	26 (5)	13 (10)
HSV-2 Positive	120 (5)	5 (1)	41 (4)	50 (9)	24 (19)
Vaginal sex reported	672 (27)	47 (6)	256 (24)	286 (52)	83 (65)
Condom use during last sex*	408 (59)	29 (58)	167 (64)	161 (55)	51 (59)
Median age of sexual debut (IQR)	16 (14–16)	-	-	-	-

* percentage calculated as the proportion of young women who used a condom at last sex, out of all young women who reported vaginal sex.

Secondary outcomes include markers of sexual risk behaviours. Slightly more than one quarter (26.6%) of all young women reported having ever had vaginal sex (increasing from 6.1% of participants aged 13–14 to 65.4% of those aged 19–20, p = <0.001). More than half (59.4%) of these young women reported condom use during their last sexual encounter. The proportion of sexually active young women who reported condom use during last sex was not significantly different across age groups (p = 0.172). Of those who reported having sex, the median age of sexual debut reported at baseline was 16 years (IQR 14–16) (Table 3).

### Exposure: Interest in education

The primary exposures relate to perceived household interest in education. Responses to the seven questions related to this subject are shown in Table 4. Mothers were by far the most commonly identified person most involved in the young woman’s education (in 69% of cases). This is compared to fathers—identified in 12.1% of cases—and grandparents, who filled this role in 8.7% of cases.

**Table 4:** Household interest in education, overall and by age group.

Household interest in education	Total (%)*	Age 13–14 (%)*	Age 15–16 (%)*	Age 17–18 (%)*	Age 19–20 (%)*
	n = 2533 (100)	n = 773 (31)	n = 1075(42)	n = 557 (22)	n = 128 (5)
Person most interested in schooling					
Mother	1744 (69)	552 (72)	734 (68)	337 (68)	81 (63)
Father	307 (12)	76 (10)	134 (12)	79 (14)	18 (14)
Grandparent	219 (9)	68 (9)	84 (8)	49 (9)	18 (14)
Older sister	112 (4)	32 (4)	54 (5)	22 (4)	4 (3)
Older brother	64 (3)	18 (2)	25 (2)	16 (3)	5 (4)
Aunt	25 (1)	8 (1)	12 (1)	4 (1)	1 (1)
Uncle	31 (1)	9 (1)	16 (1)	5 (1)	1 (1)
Cousin	8 (0.3)	3 (0.3)	3 (0.3)	2 (0.4)	0 (0)
Other–not a blood relative	19 (1)	6 (1)	11 (1)	2 (0.4)	0 (0)
School work checked					
Never	319 (13)	82 (11)	139 (13)	76 (14)	22 (17)
Sometimes	850 (34)	244 (2)	367 (34)	194 (35)	45 (35)
Always	1361 (54)	447 (58)	567 (53)	286 (51)	61 (48)
Helps with homework					
Never	373 (15)	83 (11)	141 (13)	114 (21)	35 (27)
Sometimes	1137 (45)	331 (43)	504 (47)	252 (45)	50 (39)
Always	1021 (40)	359 (46)	429 (40)	190 (34)	43 (34)
Discuss studies					
Never	271 (11)	66 (9)	104 (10)	80 (15)	21 (17)
Sometimes	1137 (47)	361 (49)	500 (48)	225 (42)	51 (41)
Always	1029 (42)	314 (42)	436 (42)	227 (43)	52 (42)
Discuss Marks					
Never	290 (12)	72 (10)	111 (11)	86 (16)	21 (17)
Sometimes	1058 (43)	320 (42)	457 (43)	232 (43)	49 (40)
Always	1122 (45)	363 (48)	487 (46)	219 (41)	53 (43)
Perceived educational goals					
Complete this year and no more	220 (9)	65 (9)	76 (7)	67 (12)	12 (0.5)
Pass matric	821 (33)	249 (33)	339 (32)	178 (33)	55 (43)
Attend university/technikon	1451 (58)	448 (59)	641 (61)	302 (55)	60 (47)
Perceived disappointment at drop out					
Not at all	631 (25)	241 (32)	233 (22.2)	121 (22)	36 (29)
Somewhat	164 (7)	53 (7)	67 (6.4)	39 (7)	5 (4)
Very much	1684 (68)	461 (61)	750 (71)	389 (71)	84 (67)

*percents may not add to 100 due to rounding

Across all six remaining variables the majority of young women reported high levels of involvement and interest in their schooling by a caregiver. Less than fifteen percent of young women reported having no supervision or assistance with homework and no discussions about studies at school or marks. A third of young women felt they were expected to complete secondary school and over half felt that the person most involved in their schooling would like them to attend a university or tertiary institution. However, a quarter of young women felt that the person most involved in their schooling would not be disappointed should they drop out of school (Table 4). No consistent pattern was identified in relation to the person identified as most interested in education and the level of interest in education.

Household educational support patterns were broken down by age (Table 4): supervision and help with homework both appear to decline with age: 57.96% of young women aged 13–14 years reported always having their school work checked, as opposed to 47.66% of 19–20 year olds. This pattern continues when considering assistance with homework, with 10.74% of 13–14 year olds never receiving help with homework, compared to 27.34% of 19–20 year olds. A similar pattern was demonstrated between age and discussion of studies (8.77% of 13–14 year olds never discussed studies compared to 16.94% of 19–20 year olds) and age and discussion of marks (9.40% of 13–14 year olds never discussed marks compared to 17.07% of 19–20 year olds). There does not appear to be a clear relationship between age and educational goals, nor between age and disappointment at dropout.

### Biological outcomes

No measure of interest in education was found to be significantly associated with HIV prevalence after adjusting for age and grade at enrollment (Table 5). (Note: for crude OR see S1 Table). However, more frequent discussion of marks was associated with greater HIV infection prevalence, with borderline significance (OR 1.39, 95%CI: 0.98–1.98, p = 0.062).

**Table 5:** Associations between household educational support and HIV, HSV-2 and Sexual risk behaviours, adjusted for age and grade at enrollment.

Exposure	HIV			HSV-2			Vaginal sex			Condom use at last sex*			Age of sexual Debut*Ŧ		
	n	OR (95% CI)	P value	n	OR (95% CI)	P value	n	OR (95% CI)	P value	n	OR (95% CI)	P value	n	Coefficient (95% CI)	P value
Check HW	2526	0.99 (0.72–1.35)	0.929	2526	0.96 (0.74–1.25)	0.778	2521	0.82 (0.72–0.94)	0.005	687	1.09 (0.88–1.34)	0.424	661	-0.02 (-0.36–0.33)	0.932
Help HW	2527	0.94 (0.69–1.29)	0.706	2527	1.10 (0.84–1.44)	0.482	2522	0.99 (0.86–1.14)	0.859	687	1.06 (0.86–1.31)	0.602	661	-0.07 (-0.43–0.28)	0.683
Discuss studies	2433	1.34 (0.94–1.91)	0.108	2433	1.06 (0.80–1.40)	0.701	2429	0.93 (0.80–1.08)	0.347	654	1.16 (0.93–1.45)	0.186	630	0.30 (-0.07–0.66)	0.110
Discuss marks	2466	1.39 (0.98–1.98)	0.062	2466	1.14 (0.86–1.51)	0.371	2462	0.82 (0.71–0.95)	0.009	662	1.22 (0.99–1.51)	0.068	638	0.28 (-0.07–0.63)	0.120
Educational goals	2488	0.89 (0.64–1.24)	0.506	2488	0.81 (0.61–1.07)	0.134	2484	0.82 (0.71–0.96)	0.011	681	1.14 (0.92–1.43)	0.235	656	-0.33 (-0.70–0.04)	0.082
Disappointment at dropout	2475	1.12 (0.85–1.47)	0.428	2475	1.09 (0.86–1.38)	0.461	2471	1.24 (1.10–1.41)	0.001	680	0.90 (0.74–1.09)	0.274	654	-0.20 (-0.52–0.13)	0.232

*This analysis is only among those young women who have had vaginal sex

^Ŧ^ This analysis is unadjusted for age or grade at enrollment given that age is included in the outcome

n refers to the total number of young women included in each logistic regression model, conducted separately for each exposure.

None of the exposures of interest were significantly associated with HSV-2 status.

### Sexual risk behaviours

After adjusting for age and grade at enrollment, the odds of having had vaginal sex were significantly lower in those who reported more regular homework supervision (OR 0.82, 95%CI: 0.72–0.94, p = 0.005), discussion of marks (OR 0.82, 95%CI: 0.71–0.95, p = 0.009) and greater educational goals (OR 0.82, 95% CI: 0.71–0.96, p = 0.011). In contrast, disappointment at dropout was significantly associated with having had vaginal sex (OR 1.24, 95%CI: 1.10–1.41, p = 0.001). Discussion of studies and assistance with homework were not associated with having had vaginal sex (Table 5).

None of the educational support variables were significantly associated with condom use or age of sexual debut (Table 5).

## Discussion

This study found high levels of educational support across all measures: over 80% of young women reported caregiver involvement with homework, studies and marks; a majority (58%) felt their caregiver would want them to attend university, and over two-thirds (68%) felt their caregiver would be very disappointed if they were to drop out of school. HIV and HSV-2 prevalence were not significantly associated with household educational support. Checking homework, discussing marks and greater educational goals were noted to have significant protective associations in reducing vaginal sex. In contrast, greater disappointment at dropout was associated with increased reports of this sexual risk behaviour.

To our knowledge, this is the first study to examine the nature of household educational support in such depth in a rural South African context and so there is limited comparative evidence. One study which looked specifically at the question of support for homework, and parental engagement with teachers and schools in previously disadvantaged black schools in South Africa reported much lower levels of engagement and support than described in this study [37]. That study further noted that low levels of parental education contributed to a lack of educational support[37]. It is therefore even more surprising that such high levels of educational support were described at baseline in this study given the relatively low levels of parental education in the Bushbuckridge community, though this result may reflect bias from selecting girls that were enrolled in school.

The baseline HIV prevalence of 3.20% was lower than the national estimate of 5.6% for females 15–19 years [1]. However, all participants in the study were in school at baseline, a protective factor which may partially account for a lower baseline HIV prevalence in this study. Regardless of the explanation, the lower HIV prevalence meant that this study was underpowered to detect associations between HIV and interest in education.

HSV-2 prevalence at baseline was 4.74%. This would appear to be significantly lower than national and regional population estimates which range from 9.0% to 40% in under 20 year olds (though there is very limited data on HSV-2 prevalence from South Africa in recent years) [38–40]. No significant associations were shown between HSV-2 and any of the exposures of interest, again likely reflecting the low prevalence rates of HSV-2 at baseline, and the fact that only a quarter of young women reported being sexually active at baseline.

Our analysis suggests that different aspects of educational support have varying effects on sexual risk behaviours, reflecting the different mechanisms by which the parent-child relationship influences adolescents’ behaviours. Checking homework and discussion of marks capture a sense of parental supervision and monitoring, which has been shown to be associated with lower rates of sexual activity, and reduced sexual risk behaviours [10–12,15,20,41]. In contrast, assistance with homework and discussion of studies might be a greater reflection of the caregiver’s own educational background. In the context of relatively low levels of education among parents, it is likely that they are simply unable to assist with homework once the young women reach a certain level of schooling.

Moreover, academic engagement in the form of assistance and discussion of school work demonstrates a different set of power dynamics to those of parental supervision or monitoring. Whilst the latter is based on a clear hierarchy in the family with potential disciplinary consequences if the young woman fails to follow established rules of the household, the former might suggest a more equal relationship with an exchange of ideas. The findings from this study suggest that whilst discussion about and assistance with studies might be useful, it does not impact on sexual risk behaviours.

The perception that caregivers have greater educational goals for their children has been shown to drive adolescent educational attainment, enhance adolescents’ own educational aspirations and promote self-esteem [36,41]. Both greater educational attainment and improved self-esteem might underpin the significant association noted in this study between greater educational goals and reduced odds of having had vaginal sex.

Finally, disappointment at dropout appears to capture a sense of negative pressure associated with adolescent rebelliousness and risky behaviour. It may be that perceived disappointment at dropout is part of disappointment in a range of behaviours which are considered undesirable, including sexual activity. Additionally, dropout from school, might be on the pathway from lack of parental engagement leading to greater sexual risk behaviours which would be in keeping with the trend demonstrated by other measures. However, given the cross-sectional design of this study it is difficult to determine whether sexual risk behaviours are seen as the cause or the consequence of school drop-out.

Condom use and age of sexual debut were not significantly associated with household educational support. This might be due, in part, to the fact that only a quarter of young women reported being sexually active at baseline (and therefore answered questions relating to condom use and age of sexual debut).

### Strengths and limitations

Key strengths of this study relate to the size of the sample, the sampling technique, the study setting and the depth of questioning. This study involved 2533 participants, a much larger sample than often studied and used population-based sampling rather than school-based sampling. The location of this study in a rural South African community is significant in diversifying the geographical location of research generated around questions of parental involvement, the parent-child relationship and its effect on adolescent behaviours. Most previous research on the topic took place in the United States of America, which may not be comparable to South African populations where family structure, cultural norms and parenting styles are different. This is especially true in the comparison of urban populations (which form the source populations for the referenced studies) to rural populations (where extended families are more prominent, and with greater emphasis on communal upbringing of children). Finally, the depth of questioning around household interest in education offers the opportunity to consider different mechanisms through which such involvement might affect sexual risk behaviours, HIV and HSV-2 status.

However, this study has a number of important limitations which should be taken into account when interpreting results: firstly, this is a cross-sectional study and so the direction of associations demonstrated cannot be determined definitively. Secondly, whilst prevalence data is valuable, especially relating to HSV-2 (as there is currently very limited data estimating HSV-2 prevalence in South African communities), prevalence fails to differentiate between vertical and horizontal transmission of disease. Although not expected to have a large impact on the prevalence figures detected in this study, it is possible that a portion of HIV and HSV-2 disease captured was not acquired during sexual intercourse, but rather from mother-to-child transmission (and therefore clearly demonstrates a different set of risk factors). Thirdly, this study was underpowered to detect associations with HIV or HSV-2 prevalence as population prevalence was lower than expected. Finally, this study relies on the self-reporting of sexual behaviours by participants. Efforts were made to encourage accurate reporting of behaviour, including the use of ACASI software; however, one cannot guarantee the reliability of reporting of sexual risk behaviours.

### Recommendations for future research and policy considerations

Further research is recommended with a study that is better powered to examine associations with HIV and HSV-2. Longitudinal studies using HIV and HSV-2 incidence rather than prevalence would enable a more reliable assessment of causality. Finally, the authors recommend that analysis of measures of educational support be replicated in urban and peri-urban settings and across income brackets.

## Conclusion

Young women in rural South Africa report experiencing high levels of household educational support across all measures. Whilst the different dimensions of educational support varied in their impact on sexual risk behaviours, this study provides evidence to suggest that household involvement in schooling, in the form of homework supervision and discussion of marks, is associated with lower odds of having had vaginal sex, though not with HIV or HSV-2 prevalence.

## Supporting information

S1 Table Crude odds ratio of the association between interest in education and HIV, HSV-2 and Sexual risk behaviours. (DOCX)

S1 Appendix Annotated case report form. (PDF)

S2 Appendix Annotated baseline questionnaire. (PDF)

S3 Appendix Codebook. (XLSX)

S1 Dataset Anonymised dataset. (XLS)
