# Supplementary material for: The association between perceived household educational support and HIV risk in young women in a rural South African community (HPTN 068): A cross sectional study
Source: PLoS One. 2019 Jan 17;14(1):e0210632. doi: 10.1371/journal.pone.0210632 (PMC6336295; doi:10.1371/journal.pone.0210632)
Supplement: S1 Table — (DOCX) [file pone.0210632.s001.docx]

**Supporting Information**

**S1 Table. Crude odds ratio of the association between interest in education and HIV, HSV-2 and Sexual Risk Behaviours.**

| **Exposure** | **HIV** | | **HSV-2** | | **Vaginal sex** | | **Condom use at last sex*** | | **Young Age of sexual debut*** | |
| --- | --- | --- | --- | --- | --- | --- | --- | --- | --- | --- |
|  | **OR (95% CI)** | **P value** | **OR (95% CI)** | **P value** | **OR (95% CI)** | **P value** | **OR (95% CI)** | **P value** | **HR (95% CI)** | **P value** |
| **Check HW** | 0.89 (0.65-1.21) | 0.466 | 0.92 (0.72-1.19) | 0.539 | 0.79 (0.70-0.89) | <0.001 | 1.10 (0.89-1.35) | 0.378 | 0.90 (0.81-1.00) | 0.048 |
| **Help HW** | 0.85 (0.62-1.16) | 0.304 | 0.91 (0.70-1.17) | 0.461 | 0.81 (0.72-0.92) | 0.001 | 1.08 (0.87-1.33) | 0.484 | 1.01 (0.91-1.13) | 0.840 |
| **Discuss studies** | 1.33 (0.92-1.92) | 0.125 | 0.95 (0.72-1.26) | 0.730 | 0.87 (0.76-0.99) | 0.048 | 1.16 (0.93-1.44) | 0.198 | 0.97 (0.86-1.09) | 0.578 |
| **Discuss marks** | 1.39 (0.97-1.99) | 0.076 | 1.05 (0.79-1.38) | 0.751 | 0.77 (0.68-0.88) | <0.001 | 1.23 (0.99-1.52) | 0.056 | 0.88 (0.79-0.98) | 0.025 |
| **Educational goals** | 0.80 (0.58-1.11) | 0.188 | 0.80 (0.61-1.04) | 0.093 | 0.81 (0.71-0.93) | 0.002 | 1.15 (0.92-1.42) | 0.218 | 0.89 (0.80-1.00) | 0.044 |
| **Disappointment at dropout** | 1.04 (0.80-1.36) | 0.755 | 1.19 (0.95-1.50) | 0.134 | 1.31 (1.17-1.46) | <0.001 | 0.91 (0.75-1.10) | 0.330 | 1.15 (1.05-1.27) | 0.004 |

*This analysis is only among those young women who have had vaginal sex
