# Supplementary material for: The association between perceived household educational support and HIV risk in young women in a rural South African community (HPTN 068): A cross sectional study
Source: PLoS One. 2019 Jan 17;14(1):e0210632. doi: 10.1371/journal.pone.0210632 (PMC6336295; doi:10.1371/journal.pone.0210632)
Supplement: S1 Appendix — (PDF) [file pone.0210632.s002.pdf]

Participant ID ptid

Site Number – Participant Number – Chk – Cohort

Demographics: Young Woman

DEMstid
Unit/Site ID

Form Completion Date DEMdt
dd MMM yy

1. What is the participant's (young woman) date of birth? .... DEMbthdt dd MMM yy → *If unknown, record age:* DEMage years

2. What is your gender? .... male DEMsex female

NOT APPLICABLE FOR THIS PROTOCOL.

3. Mark the appropriate racial category for this participant.

DEMblack 3a. Black

DEMclrd 3b. Coloured

DEMwhite 3c. White

DEMindn 3d. Indian

DEMraco 3e. other, specify: DEMracox

Visit Code visit .

Participant ID

ptid - - -

Site Number | Participant Number | Chk | Cohort

Demographics: Parent/Guardian

Form Completion Date

DEPdt

dd | MMM | yy

1. What is the Parent/Guardian's Participant ID? .... DEPpgpid - - -

Site Number | Participant Number | Chk | Cohort

2. What is the Parent/Guardian's date of birth? DEPbthdt (dd MMM yy) → *If unknown, record age:* DEPage *years*

3. What is the Parent/Guardian's gender?..... *male* DEPsex *female*

4. Mark the appropriate racial category for the Parent/Guardian.

DEPblack 4a. Black

DEPclrd 4b. Coloured

DEPwhite 4c. White

DEPindn 4d. Indian

DEPraco 4e. other, specify: DEPracox ______________________

5. Did the Parent/Guardian complete the Baseline Household Questionnaire? .... *yes* DEPpghhq *no*

formlang Language

sfdt_002 Staff Initials / Date

Participant ID

ptid - - -

Site Number | Participant Number | Chk | Cohort

Enrollment

Enrollment Date

ENRdt

dd MMM yy

1. Did the participant complete the ACASI Baseline Questionnaire? .... *yes* *no* ENRacasi

2. Mark the village (village number) where the participant has her permanent residence:

ENRagin Agincourt (101)	ENRjust Justicia A/Justicia B (109)	ENRxan Xanthia (117)
ENRclb Croquet Lawn B (102)	ENRkila Kildare A (110)	ENRkhay Khaya Lami (118)
ENRcl Croquetlawn (103)	ENRkilbc Kildare B/Kildare C (111)	ENRdmpha Dumphries A (119)
ENRcunna Cunningmore A (104)	ENRlilla Lillydale A/Rholane (112)	ENRdmphb Dumphries B (120)
ENRcunnb Cunningmore B (105)	ENRlillb Lillydale B (113)	ENRdmphc Dumphries C (121)
ENRhunt Huntington (106)	ENRnewtb Newington B (114)	ENRirec Ireagh C (122)
ENRirea Ireagh A (107)	ENRnewtc Newington C (115)	ENRsmstc Somerset C (123)
ENRireb Ireagh B (108)	ENRsmsta Somerset A/Somerset B (116)	ENRblfst Belfast (124)
ENRmerry Merrypebble Stream (125)	ENRcork Cork (126)	ENRthul Thulamahashe (127)
ENRrolle Rolle C (125)		
ENRkhum Khumani (125)		

3. What is the grade of the participant at enrollment? .... *8th grade* *9th grade* *10th grade* *11th grade* ENRgrade

4. Was informed consent for long-term specimen storage signed? .... *yes* *no* ENRspec

5. Record the Randomization Envelope Number: .... ENRren

6. Indicate the participant's randomization assignment: .... *intervention (conditional cash transfer)* *control (no payments)* ENRra

formlang Language

sfdt_100 Staff Initials / Date

Participant ID: ptid - - -
Site Number | Participant Number | Chk | Cohort

Visit Date: FUVdt (dd MMM yy)

Follow-up Visit

1. Did the participant complete the ACASI Follow-up Questionnaire? .... yes / no — FUVacasi

2. Since the last visit, has the participant experienced any social harms as a result of participation in this study? yes / no — FUVsh
   If yes, complete Social Impact Log.

3. Since the last visit, did the participant change her village of permanent residence? .... yes / no — FUVvilch
   If no, go to item 4.

   3a. Record new Village Number: .... FUVvilln
   If 125, specify village: FUVvills
   - Merrypebble Stream
   - Rolle C
   - Khumani

4. Since the last visit, has there been a change in the participant's Parent/Guardian of record? .... yes / no — FUVpgch
   If no, go to item 5.

   4a. Was a Demographics: Parent/Guardian CRF submitted for this parent/guardian at a previous visit? yes / no — FUVpgdep
   If no, complete Demographics: Parent/ Guardian. Go to item 5.

   4b. Record Parent/Guardian ID: .... FUVpgpid - - -
   Site Number | Participant Number | Chk | Cohort

5. Did the Parent/Guardian complete the Follow-up Household Questionnaire? .... yes / no — FUVpghq

Participant ID

ptid – – –

Site Number | Participant Number | Chk | Cohort

Virology Test Results

Initial Specimen Collection Date

VTRdt

dd MMM yy

1. **Specimen Collection for Storage**

	stored	not stored	reason not stored:
1a. Plasma	VTRpls	☐ →	VTRplsr
1b. Dried blood spot	VTRdbs	☐ →	VTRdbpr

VTRhiv *Mark this box if participant was previously confirmed HIV+. ~~End of form.~~ End of form.*

Not done/ Not collected	Alternate Collection Date dd	MMM	yy		negative	positive	
VTRhsvn	VTRhsvdt			2. ~~HSV-2 Result~~ No longer recorded on the VTR-1. See HSV-1 form.	VTRhsv	☐	VTRhsv

3. **HIV Test Results**

Not done/ Not collected	Alternate Collection Date dd	MMM	yy		kit code	negative	positive	
VTRrapn	VTRrapdt			3a. Rapid test 1	VTR1rt	VTRrap	☐	VTRra
VTR2rapn	VTR2rpdt			3b. Rapid test 2	VTR2rt	VTR2rap	☐	VTR2r

If one or both are positive, complete HIV Confirmatory Test Results.

Comments: VTRcomm

formlang
Language

sfdt_131
Staff Initials / Date

Participant ID

ptid – – –

Site Number | Participant Number | Chk | Cohort

HSV-2 Test Result

Specimen Collection Date

HSVdt

dd *MMM* *yy*

		negative	*positive*	*not done*	
1.	**HSV-2 Result**	☐ HSVhsv	☐	☐ →	***If not done, specify reason in Comments.***

Comments: HSVcomm ______________________________

____

____

formlang

Language

sfdt_135

Staff Initials / Date

Participant ID

ptid - - -

Site Number | Participant Number | Chk | Cohort

HIV Confirmatory Test Results

Sample 1

1. Absolute CD4+ HTRcd4 cells/mm3

2. HIV Western Blot .... HTRwb1 negative / positive / indeterminate

If negative or indeterminate, contact Network Lab.

If positive, go to Sample 2: item 3.

3. Sample 2 .... Not done/ Not collected HTR2nd

Specimen Collection Date (dd MMM yy) HTR2cldt

If not done/not collected, go to Sample 3: Item 4.

3a. HIV Western Blot HTRwb2 negative / positive / indeterminate

If negative or indeterminate, contact Network Lab.

3b. Plasma Storage .... HTR2pls stored / not stored → Reason: HTR2plsx

3c. Dried Blood Spot Storage .... HTR2dbs stored / not stored → Reason: HTR2dbsx

4. Sample 3 .... Not done/ Not collected HTR3nd

Specimen Collection Date (dd MMM yy) HTR3cldt

If not done/not collected, go to item 5.

4a. HIV Western Blot HTRwb3 negative / positive / indeterminate

If negative or indeterminate, contact Network Lab.

4b. Plasma Storage .... HTR3pls stored / not stored → Reason: HTR3plsx

4c. Dried Blood Spot Storage .... HTR3dbs stored / not stored → Reason: HTR3dbsx

5. Final HIV status: .... HTRfin negative / positive / other, specify: HTRfinx

Comments: HTRcomm

formlang Language

sfdt_145 Staff Initials / Date

Participant ID

ptid - - -

Site Number | Participant Number | Chk | Cohort

Social Impact Log

1. Concisely describe social impact related to study participation experienced by the participant:

SILsumx

2. Onset date: .... SILondt (dd MMM yy)

3. Reported at visit:..... SILatvis .

4. Social impact code: SILcode

5. Social impact grade: SILgrade
   - Grade 1: mild
   - Grade 2: moderate
   - Grade 3: severe
   - Grade 4: potentially life-threatening

Social Impact Codes: *See back for definitions.*

01 Personal Relationships
02 Travel/Immigration
03 Employment
04 Education
05 Medical/Dental
06 Health Insurance
07 Life Insurance
08 Housing
09 Military/Other Government Agency
10 Other
11 Harassment, bullying, coercion
12 Violence
13 Sexual

Visit Code visit .

Participant ID

ptid - - -

Site Number | Participant Number | Chk | Cohort

Follow-up CD4 Test Result

Specimen Collection Date

FCDdt

dd MMM yy

1. Absolute CD4+ FCDcd4 cells/mm3 not done FCDcd4n → *If not done, specify reason in Comments.*

Comments: FCDcomm

formlang Language

sfdt_155 Staff Initials / Date

Participant ID

ptid - - -

Site Number | Participant Number | Chk | Cohort

Graduation Visit

Visit/Form Completion Date

GVdt

dd MMM yy

GVlav ► ***Mark this box if visit was completed and was a Limited Assessment. Complete LAV-1, ESI-1, and TM-1 (mark reason 2a). End of form.***

1. Did the participant complete this visit?

   GVcmplt yes → ***If yes, complete VTR-1, ESI-1, and TM-1 (mark reason 2a). Complete HSV-1 and/or FCD-1, if applicable. End of form.***

   ☐ no → ***If no, complete TM-1 and ESI-1.***

   1a. If no, indicate reason. ***Mark only one.***

   GVrsn participant refused

   ☐ participant lost to follow-up

   ☐ previous visit was too recent

   ☐ participant deceased

   ☐ other, specify: GVrsnotx ____________________

Comments: GVcomm ____________________

formlang Language

sfdt_220 Staff Initials / Date

Participant ID: ptid - - - (Site Number, Participant Number, Chk, Cohort)

Post-intervention Visit

Visit/Form Completion Date: PIVdt (dd MMM yy)

1. Was the post-intervention visit completed? PIVcmplt yes / no — *If yes, go to item 2.*

   1a. If no, why wasn't the visit completed? ***Mark only one. End of form.*** PIVrsn
   - unable to contact participant
   - participant refused visit
   - participant incarcerated
   - participant deceased
   - investigator decision, specify: PIVrsnid
   - other, specify: PIVrsnox

2. Was a limited assessment visit conducted at this post-intervention visit? PIVlav yes / no — ***If yes, complete the Post-Intervention Limited Assessment Visit form.***
3. Did the participant complete the ACASI Young Woman Questionnaire? PIVacasi
4. Did the participant complete the Household Questionnaire? PIVhhq
5. Since the last visit, has the participant experienced any social harms as a result of participation in this study? PIVsh — ***If yes, complete Social Impact Log.***
6. Since the last visit, did the participant change her village of permanent residence? PIVvilch yes / no — ***If no, go to item 7.***

   6a. Record new Village Number: PIVvilln — ***If 125, specify village:*** PIVvills
   - Merrypebble Stream
   - Rolle C
   - Khumani

7. Blood Pressure:

   7a. Blood Pressure–1st measurement: PIVbp1s / PIVbp1d mmHg OR PIVbp1n Not done

   7b. Blood Pressure–2nd measurement: PIVbp2s / PIVbp2d mmHg OR PIVbp2n Not done

   7c. Blood Pressure–3rd measurement: PIVbp3s / PIVbp3d mmHg OR PIVbp3n Not done

8. Height: .... PIVht . cm OR PIVhtn Not done
9. Weight: .... PIVwt . kg OR PIVwtn
10. Waist Circumference: .... PIVwst . cm OR PIVwstn
11. BMI: .... PIVbmi . kg/m^2 OR PIVbmin
12. Was informed consent for long-term specimen storage signed? .... PIVspec yes / no

Comments: PIVcomm

bit4b288b282b281_230 24-MAR-15

Language: formlang

Staff Initials / Date: sfdt_230

Visit Code: visit

HPTN 068 (183) LAV-1 (250)

Participant ID: ptid
Site Number – Participant Number – Chk – Cohort

Limited Assessment Visit

Initial Specimen Collection Date: LAVdt (dd MMM yy)

1. Were HIV rapid tests performed by finger stick? .... yes: LAVrap / no → *If no, do not complete form. Do not fax to SCHARP DataFax.*

1a. Dried blood spot storage .... stored: LAVdbs / not stored → Reason not stored: LAVdbsr

1b. HIV Test Results

	kit code	negative	positive
1b1. Rapid test 1	LAVkit1	LAVrap1	☐
1b2. Rapid test 2	LAVkit2	LAVrap2	☐

Alternate Collection Date: LAVhivdt (dd MMM yy)

2. Was a venous blood draw done? yes: LAVvbd / no → *If no, end of form.*

2a. HIV Western Blot .... negative: LAVwb / positive ☐ / indeterminate ☐ → *If negative or indeterminate, contact Network Lab.*

2b. Plasma storage .... stored: LAVpls / not stored → Reason not stored: LAVplsr

Comments: LAVcomm

Language: formlang

Staff Initials / Date: sfdt_250

Plate Number: 260 PLV-1: Post-intervention Limited Assessment Visit

Visit Code: visit

HPTN 068 (183) (PLV-1) 260

Participant ID: ptid
Site Number - Participant Number - Chk - Cohort

Post-intervention Limited Assessment Visit

Initial Specimen Collection Date: PLVdt (dd MMM yy)

1. Were HIV rapid tests performed by finger stick? .... yes: PLVrap / no → *If no, do not complete form. Do not fax to SCHARP DataFax.*

1a. Dried blood spot storage .... stored: PLVdbs / not stored → Reason not stored: PLVdbsr

1b. HIV Test Results

	kit code	negative	positive
1b1. Rapid test 1	PLVkit1	PLVrap1	
1b2. Rapid test 2	PLVkit2	PLVrap2	

Alternate Collection Date (dd MMM yy): PLVhivdt

2. Was a venous blood draw done? yes: PLVvbd / no → *If no, end of form.*

2a. HIV Western Blot .... negative: PLVwb / positive / indeterminate — *If negative or indeterminate, contact Network Lab.*

2b. Plasma storage .... stored: PLVpls / not stored → Reason not stored: PLVplsr

Comments: PLVcomm

bit4_260 bit3_260 bit2_260 bit1_260 30-JAN-15

\\READYNAS\clients\HPTN\HPTN068\folder from SCHARP\PTN_068\forms\DF-Net Frame 9 CRFs\p068_PLV.fm

Language: formlang   Staff Initials / Date: sfdt_260

Participant ID

ptid - - -

Site Number | Participant Number | Chk | Cohort

Interim Visit

Visit Date

IVdt

dd MMM yy

1. What is the reason for this interim visit? *Mark all that apply.*

IVpgch 1a. change in Parent/Guardian

1a1. Was a Demographics: Parent/Guardian CRF submitted for this parent/guardian at a previous visit? .... yes IVpgdep no → ***Complete Demographics: Parent/Guardian. Go to item 1b.***

1a2. Record Parent/Guardian ID: .... IVpgpid - - -

Site Number | Participant Number | Chk | Cohort

IVvilch 1b. change in participant's village of permanent residence

1b1. Record new Village Number: .... IVnvill

If 125, specify village:

IVvills Merrypebble Stream

Rolle C

Khumani

IVsh 1c. to report social harm(s) → ***Complete Social Impact Log.***

IVhiv 1d. HIV testing → ***Complete Virology Test Results.***

IVcd4 1e. follow-up CD4 testing → ***Complete Follow-up CD4 Test Results.***

IVhsv 1f. HSV testing → ***Complete HSV-2 Results.***

IVoth 1g. other, specify: IVothx ____________________

formlang Language

sfdt_350 Staff Initials / Date

Participant ID

ptid - - -

Site Number | Participant Number | Chk | Cohort

Missed Visit

Form Completion Date

MVfcdt

dd MMM yy

1. Target Visit Date: MVtdt (dd MMM yy)

2. Reason visit was missed. *Mark only one.*

- MVreasn 2a. unable to contact participant
- 2b. unable to schedule appointment(s) within target window
- 2c. participant refused visit
- 2d. participant incarcerated
- 2e. participant admitted to a health care facility
- 2f. participant withdrew from the study → ***Complete a Termination form.***
- 2g. participant deceased → ***Complete a Termination form.***
- 2h. other, specify:

MVotrex

Comments: MVcomm

HPTN 068 (183) PIL-1 (487)

Participant ID

ptid - - -

Site Number | Participant Number | Chk | Cohort

Participant Incident Log — For Internal Use Only

dd MMM yy

Form Completion Date: PILdt

Study Project Manager: PILstpm

If applicable: **Plate #:** PILplate

Visit/Page #: PILvisit

Incident: *Mark all that apply*

- PILenrol inappropriate enrollment/ineligible
- PILrand randomization
- PILhivts HIV testing
- PILprotd protocol deviation/event
- PILinco other, specify: PILincox
- PILprots [protocol-specific reason]

Details: *Provide additional information, including date(s), site(s), lab(s), outcome, etc. as appropriate.*

PILdtlsx

FOR INTERNAL USE ONLY

Participant ID

ptid - - -

Site Number | Participant Number | Chk | Cohort

End of Study Inventory

Form Completion Date

ESIdt

dd MMM yy

1. What is the **highest** visit code (scheduled or interim) for this participant, recorded on a form submitted via DataFax?...

   visit code ESIlvis

2. How many interim visits were conducted for this participant during the study and recorded on a form submitted via DataFax? ....

   # of interim visits ESIivis

3. Indicate the **highest** page number submitted for this participant for the Social Impact Log:

   3a. Social Impact Log (SIL-1) ....

   page # ESIsilpg **OR** *no pages submitted* ESIsilpn

Comments: ESIcomm

formlang
Language

sfdt_489
Staff Initials / Date

Termination

Participant ID

ptid – – –

Site Number | Participant Number | Chk | Cohort

1. Termination Date: TMtmdt (dd MMM yy) *Date the site determined that the participant was no longer in the study.*

2. Reason for termination. *Mark only one.*

   - TMtrmrsn [illegible] scheduled exit visit/end of study ⟶ ***End of form.***
   - 2b. death, *indicate date and cause if known*
     - 2b1. date of death TMddt (dd MMM yy) **OR** TMddun date unknown
     - 2b2. cause of death TMdcau **OR** TMdcun cause unknown
   - 2c. participant refused further participation, specify: TMrefrx
   - 2d. participant unable to adhere to visit schedule
   - 2e. participant relocated, no follow-up planned
   - 2f. investigator decision, specify: TMinvdx
   - 2g. unable to contact participant
   - 2h. HIV infection — **NOT APPLICABLE FOR THIS PROTOCOL.**
   - 2i. inappropriate enrollment
   - 2j. invalid ID due to duplicate screening/enrollment
   - 2k. other, specify: TMtrmox
   - 2l. early study closure

Comments: TMcomm

formlang — Language

sfdt_490 — Staff Initials / Date
