# Supplementary material for: The association between perceived household educational support and HIV risk in young women in a rural South African community (HPTN 068): A cross sectional study
Source: PLoS One. 2019 Jan 17;14(1):e0210632. doi: 10.1371/journal.pone.0210632 (PMC6336295; doi:10.1371/journal.pone.0210632)
Supplement: S2 Appendix — (PDF) [file pone.0210632.s003.pdf]

HPTN 068 Study

Young Women

Baseline Questionnaire

Survey Administered:

March 5th, 2011-December 17th, 2012

HPTN 068 Study
Young Women's Follow-Up Baseline Questionnaire

GENERAL

Which study group is the young woman in? **ARM**	5678 = Intervention (cash)<br>1234 = Control (no cash)
Young Woman De-identify ID Number: **UID**	10 - 126800
Visit Number **VISIT**	201 = Baseline<br>301 = Follow Up Year 1<br>401 = Follow Up Year 2<br>501 = Follow Up Year 3
Interview Date (ACASI variable) **TODAY**	[ ]
Interviewer initials: **INTID**	[ ]
Interview Elapsed Time **INT_TIME**	[ ]

DEMOGRAPHICS

Interviewer script: *Thank you for participating in our study. We would like to ask you questions about many aspects of your life. At any time, you may refuse to answer a question if you are not comfortable with the question. We will start by getting some basic information about you and your household.*

1.	Is your mother alive? **G1B5**	1 = Yes<br>2 = No --> **Skip to #7**<br>.D = Don't Know --> **Skip to #8**
2.	Where does your mother live now? --> **Skip to #8**<br>**G1B6 G1B6T**	1 = Your household<br>2 = Household elsewhere in Bushbuckridge<br>3 = Other urban area in South Africa<br>4 = Other rural area in South Africa<br>5 = Mozambique<br>6 = Swaziland<br>.D = Don't Know
3.	How old were you when your mother died?<br>**G1B7 G1B7T**	[ ] years<br>.D = Don't Know
4.	Is your father alive?<br>**G1B8**	1 = Yes<br>2 = No --> **Skip to #10**<br>.D = Don't Know --> **Skip to #11**
5.	Where does your father live now? --> **Skip to #11**<br>**G1B9 G1B9T**	1 = Your household<br>2 = Household elsewhere in Bushbuckridge<br>3 = Other urban area in South Africa<br>4 = Other rural area in South Africa<br>5 = Mozambique<br>.D = Don't Know
6.	How old were you when your father died?<br>**G1B10 G1B10T**	[ ] years<br>.D = Don't Know
7.	What is the highest level of education that your mother successfully completed? **G1B11 G1B11T**	1 = No school<br>2 = Some primary<br>3 = Completed primary<br>4 = Some high school<br>5 = Completed high school<br>6 = University or technikon<br>.D = Don't Know

8.	What is the highest level of education that your father successfully completed? **G1B12**	1 = No school<br>2 = Some primary<br>3 = Completed primary<br>4 = Some high school<br>5 = Completed high school<br>6 = University or technikon<br>.D = Don't Know
9.	Who is your primary caregiver? That is, who is the adult that is most responsible for taking care of you and signed the consent for you to be in this study?<br>**G1B13 G1B13T**<br><br>*Pick one response.*	1 = Mother<br>2 = Father<br>3 = Older brother and sister<br>4 = Aunts and/or uncles<br>5 = Grandparents<br>6 = Cousins<br>7 = Other adults who are not blood relatives<br>8 = I don't have a secondary caregiver<br>9 = Other family members
10.	Where were you born? **G1B14**	1 = South Africa<br>2 = Zimbabwe<br>3 = Mozambique<br>4 = Other, specify<br>5 = Swaziland<br>.D = Don't know
11.	Have you moved households within the past 12 months?<br>**G1B15**	1 = Yes<br>0 = No --> **Skip to #17**
12.	Which of the following describes your move? **G1B16**	1 = You moved on your own<br>2 = You moved with your parents<br>3 = You moved with family members not your parents<br>4 = You moved with primary caregiver that is not your relative
13.	On average, how many nights do you sleep at home every week? **G1B17**	[ ] nights

CRF Enrollment Date	**ENROLL**

<u>EDUCATION</u> – NIDS Adult, MIT Kenya, AddHealth, and NELS

Interviewer script: *Now we would like to ask you some questions about your schooling and your feelings about education.*

1.	In what year did you first attend Grade 1?<br>**FIRSTGR**	Year [___|___|___|___]
2.	Were there any grades at school that you repeated?<br>**REPEAT**	1 = Yes<br>0 = No --> **Skip to #4**
3.	What grade(s) did you repeat?<br>**GRADE1-GRADE10, GRADET**	1 = Yes<br>0 = No
4.	What is the name of the school in which you are currently enrolled? **G1C4** **G1C4T**	**See list of schools**
5.	In what grade are you currently enrolled? **G1C5**	1 = Grade 8<br>2 = Grade 9<br>3 = Grade 10<br>4 = Grade 11<br>5 = Grade 12<br>6 = University<br>.D = Don't know
6.	What is the main reason that you go to school?<br><br>*Pick only one response.*<br><br>**ATTEND**	1 = Young people are required to go to school<br>2 = Family expectations<br>3 = The subjects are interesting<br>4 = You get a feeling of satisfaction from doing well in class<br>5 = Education is important for getting a job later<br>6 = You have nothing better to do<br>7 = You play on a team or belong to a club<br>8 = School is a place to meet your friends<br>9 = Your teachers care about you and expect you to succeed in school<br>10 = To get food
7a.	On a typical weekday during the school term, how much time do you spend on homework?<br>**HOMEWKX**	1 = Hours/minutes<br>2 = Did not receive homework
7b.	On a typical weekday during the school term, how many hours/minutes do you spend on homework?<br>**HOMEWKH**	[ ] hours/minutes
8.	Did you attend primary or high school at any time during 2010/2011? **GOSCHOOL**	1 = Yes --> **Skip to #10**<br>2 = No
9.	What was the main reason you were never enrolled in school during 2010/2011?<br><br>*Pick only one response.*<br><br>**NOSIGN, NOSIGNT**<br>**Skip to #17**	1 = You fell pregnant or had a baby<br>2 = You became sick or disabled<br>3 = You acquired all the education you wanted<br>4 = No money for fees or uniform<br>5 = You were attending initiation<br>6 = You don't like or are not interested in school<br>7 = You were not doing well in school/failed too many times<br>8 = You were expelled<br>9 = You thought you were too old to continue<br>10 = Your parents told you to stop going to school<br>11 = Your boyfriend told you to stop going to school<br>12 = You had to help at home<br>13 = Caring for your own or other child

		14 = You had a problem with a teacher or school official<br>15 = You had a problem with other students<br>16 = Poor quality teaching<br>17 = Teachers often absent<br>18 = School too dangerous for girls<br>19 = School conflicted with your beliefs<br>20 = School too far from home<br>21 = Education is more important for boys than girls<br>22 = Other, specify<br>23 = Funeral/death in family<br>24 = Getting ID book or other official business
10.	What was your overall mark for the 2010/2011 school year? **G1C10 G1C10T**	1 = >80%<br>2 = 70-79<br>3 = 60-69<br>4 = 50-59<br>5 = 40-49<br>6 = 30-39<br>7 = 20-29<br>8 = <20<br>.D = Don't Know
11.	In a typical month during the 2011 school year, how many school days did you miss for any reason? **G1C6**	0 = None --> **Skip to #14**<br>1 = 1-2 days<br>2 = 3-4 days<br>3 = 5-6 days<br>4 = 7 or more days
12.	What was the main reason for your absences? **G1C7, G1C7T**<br><br>*Pick only one response.*	1 = You fell pregnant or had a baby<br>2 = You became sick or disabled<br>3 = You acquired all the education you wanted<br>4 = No money for uniform<br>5 = You were attending initiation<br>6 = You don't like or are not interested in school<br>7 = You were not doing well in school/failed too many times<br>8 = You thought you were too old to continue<br>9 = Your parents told you to stop going to school<br>10 = Your boyfriend told you to stop going to school<br>11 = You had to help at home<br>12 = Caring for your own or other child<br>13 = You had a problem with a teacher or school official<br>14 = You had a problem with other students<br>15 = Poor quality teaching<br>16 = Teachers often absent<br>17 = School too dangerous for girls<br>18 = School conflicted with your beliefs<br>19 = School too far from home<br>20 = Education is more important for boys than girls<br>21 = Other (specify)<br>22 = Funeral/death in family<br>23 = Getting ID book or other official business<br>24 = Family matters<br>25 = Visiting, vacation, school holidays<br>26 = Social worker/orphan meeting<br>27 = Buy uniform<br>28 = Checkup/dentist<br>29 = Weather<br>30 = Strike<br>31 = Playing soccer

20.		32 = Money problem<br>33 = Late/missed bus<br>34 = Went to town<br>35 = Changing schools
13.	Which of the following happened after your absences?<br><br>*Pick all that apply.*<br>1 = Yes<br>0 = No	The school did not do anything **G1C13A**<br>Someone from the school phoned or visited my house **G1C13B**<br>I was given a letter to bring home **G1C13C**<br>The school made me talk to the principal or other staff member **G1C13D**<br>School action, other **G1C13T**
14.	In the 2011 school year, who paid for your educational expenses (i.e. school uniforms transport to and from school, school meals)?<br><br>*Pick all that apply.*<br><br>1 = Yes<br>0 = No	1 = Mother **G1C8A**<br>2 = Father **G1C8B, G1C8BT**<br>3 = Grandparent(s) **G1C8C**<br>4 = Other family member **G1C8D**<br>5 = Someone outside your family/household **G1C8E**<br>6 = Boyfriend/partner **G1C8F**<br>7 = Myself **G1C8G**<br>8 = No one paid **G1C8H**<br>9 = Other (specify) **G1C8I, G1C8T**
15.	In the 2010/2011 school year, how did you get to school? **G1C15**	1 = You walked<br>2 = You got a free ride to school<br>3 = You or your family paid for a ride to school<br>4 = Other (specify)<br>5 = Bicycle<br>6 = Transport
16.	In the 2010/2011 school year, about how long did it take you to get to school in each direction? **G1C16**	1 = Less than 15 minutes<br>2 = 16-30 minutes<br>3 = 31-60 minutes<br>4 = More than an hour
17.	We know that there are sometimes problems in schools. Think now about your current school. Which of these do you think apply?<br><br>*Choose all that apply.*<br><br>1 = Yes<br>0 = No	Not enough textbooks **G1C17A**<br>Dirty classrooms **G1C17B**<br>Crowded classrooms **G1C17C**<br>Teacher often absent from class **G1C17D**<br>Teachers drunk **G1C17E**<br>Teachers being threatened by students **G1C17F**<br>Noisy classrooms **G1C17G**<br>Drug dealing **G1C17H**<br>School environment unsafe **G1C17I**<br>Sexual harassment by students **G1C17J**<br>Sexual harassment by teachers/staff **G1C17K**<br>Bullying by students **G1C17L**<br>Violent teachers **G1C17M**<br>Quality of teachers is poor **G1C17N**<br>None of the above **G1C17O**
18.	During your last full year in school, how often did you have trouble getting along with your teachers? **G1C18**	1 = Never<br>2 = Just a few times<br>3 = Regularly, about once a week<br>4 = Everyday
19.	During your last full year in school, how often did you have trouble paying attention in class? **G1C19**	1 = Never<br>2 = Just a few times<br>3 = Regularly, about once a week<br>4 = Everyday
20.	During your last full year in school, how often did you have trouble finishing your school work? **G1C20**	1 = Never<br>2 = Just a few times<br>3 = Regularly, about once a week<br>4 = Everyday

21.	During your last full year in school, how often did you have trouble getting along with other students? **G1C21**	1 = Never<br>2 = Just a few times<br>3 = Regularly, about once a week<br>4 = Everyday
22.	During your last full year in school, how often did you experience bullying from other students? **G1C22**	1 = Never<br>2 = Just a few times<br>3 = Regularly, about once a week<br>4 = Everyday
23.	During your last full year in school, how often did you have trouble with violence from other students at school? **G1C23**	1 = Never<br>2 = Just a few times<br>3 = Regularly, about once a week<br>4 = Everyday
24.	During your last full year in school, how often did you have trouble with violence from teachers/school administrators at school? **G1C24**	1 = Never<br>2 = Just a few times<br>3 = Regularly, about once a week<br>4 = Everyday
25.	During your last full year in school, how often did you get in trouble for being late for school? **G1C25**	1 = Never<br>2 = Just a few times<br>3 = Regularly, about once a week<br>4 = Everyday
26.	During your last full year at school, did you feel close to the people at your school? **G1C26**	1 = Yes<br>0 = No
27.	During your last full year at school, did you feel like you were a part of your school? **G1C27**	1 = Yes<br>0 = No
28.	During your last full year at school, were you happy to be at your school? **G1C28**	1 = Yes<br>0 = No
29.	During your last full year at school, did you feel that the teachers at your school treat students fairly? **G1C29**	1 = Yes<br>0 = No
30.	Would you like to continue at school until matric? **G1C30**	1 = Yes<br>0 = No
31.	Would you like to continue studying after passing matric, that is, after leaving school? **G1C31**	1 = Yes<br>0 = No
32.	In your opinion, what is the chance that you will be in school next year? **G1C32**	1 = No chance<br>2 = Some chance<br>3 = A high chance
33.	In your opinion, what is the chance that you will remain until matric? **G1C33**	1 = No chance<br>2 = Some chance<br>3 = A high chance
34.	Do you think it's important to be educated? **G1C34**	1 = Extremely important<br>2 = Sort of important<br>3 = Not very important<br>4 = Not at all important --> **Skip to #36**
35.	Tell us why you think it is important to be educated.<br><br>*Pick all that apply.*	You will have more choice of jobs **G1C35A**<br>You will make more money **G1C35B**<br>You will be able to run your family business more profitably **G1C35C**

	0 = No<br>1 = Yes	You will be more respected **G1C35D**<br>You will be more confident **G1C35E**<br>You will have less chance of getting HIV **G1C35F**<br>You will be able to find a better husband **G1C35G**<br>You will be a better mother **G1C35H**<br>You will be able to help parents, family **G1C35J**<br>You will have a good future **G1C35K**<br>Other, specify **G1C35I, G1C35T**
36.	Do you agree with this statement? "An education is more important for a boy than for a girl."<br>**G1C36**	1 = You agree<br>2 = You disagree<br>.D = You don't know
37.	Do you agree with this statement? "There are jealousies between girls at my school.<br>**G1C37**	1 = You agree<br>2 = You disagree<br>.D = You don't know

PARTNER GRID -- **From HPTN 062, MIT Kenya and RHRU National Survey**

Interviewer script: *We would now like to ask you some questions about relationships and your sexual history, and we will have to ask you very detailed descriptions of what we mean by some questions. These questions are not meant to make you feel uncomfortable. We need to ask about these things so that we can get as much information as possible about the experiences of young women today. All of your answers will be kept confidential. If there is a question that you really do not feel comfortable answering, please let us know and we can skip that question. We really appreciate you sharing as much information with us as possible.*

1.	Do you currently have a boyfriend or main partner? **G1E1**	1 = Yes<br>0 = No
2.	Have you ever had vaginal sex? By sex I mean when a man inserts his penis into a woman's vagina. This includes sex that was voluntary or involuntary. **G1E2**	1 = Yes<br>0 = No
3.	Have you ever had anal sex? By anal sex I mean when a man inserts his penis into a woman's anus. This includes sex that was voluntary or involuntary. **G1E3**	1 = Yes -->If Yes to #3 and No to #2, **skip to #5**<br>0 = No --> If No to #3 and Yes to #2, answer #4 and **skip #5**

Coding instructions: If ppt answered NO to Questions 1, 2, AND 3, END OF FORM

4.	How old were you when you first had vaginal sex? **G1E4_C**	[ ] years
5.	How old were you when you first had anal sex? **G1E5_C**	[ ] years
6.	In total, how many people have you had sex with in your lifetime? Even if you only had sex with a person one time, or had sex when you did not want to, we would like you to count these partners. **G1E6_C**	|___| ___| ___| partners
7.	In total, how many people have you had sex with in the past three months? **G1E7_C**	|___| ___| ___| partners<br>*If 0 partners, **skip to #12***
8.	In the past three months, how many times have you had vaginal sex? By times, we mean each individual sex act or round. If you do not remember the exact number, please make your best guess. **G1E8_C**<br><br>*If "0" skip to #10*	*If 0 partners, **skip to #10***
9.	Of these times you had vaginal sex, how many of these times did you use a condom?<br>**G1E9_C**	[ ]

Coding instructions: If ppt answered NO to Question 3 , skip to #12

10.	In the past three months, how many times have you had anal sex with someone? By times, we mean each individual sex act or round. If you do not remember exactly, please make your best guess.<br>**G1E10_C**<br><br>*If "0" skip to #12*	*If 0 partners, **skip to #12***

11.	Of these times you had anal sex, how many of these times did you use a condom?<br>**G1E11_C**	[ ]
12.	*Interviewer script: Now I would like to ask you about your sexual activity in the past year, that is, in the past 12 months. Some people may have had sex with no partners, and some may have had sex with many partners. Remember that your answers are confidential and there are not right or wrong answers to these questions.*<br>In total, how many people have you had sex with in the past 12 months?<br>**G1E12_C**	|___|___|
**Interviewer script--repeat for up to 3 partners, according to number listed in #6**		
Interviewer script: Now I will ask you about the people that you have had sex with most recently. I would like you to list your 3 most recent sex partners starting with your most recent partner. This may include a boyfriend or a short-term sex partner, or anyone with whom you have had a sexual relationship. If you have had more than one other sex partner, we will talk about up to three of your most recent sex partners. You only will identify partners by the initials that you tell me. Remember that all of your answers will be kept confidential.		
**Coding instructions: If ppt answered YES to Question 1 AND NO to Question 2 AND 3, read the following interviewer script and ask "Sex or no sex" questions only.**		
Interviewer script: If a partner is the same as the last time you completed this survey, please use the same initials as the last time		
13.	What are this partner's initials?<br>**G1E13A, G1E13B, G1E13C, G1E13F**	|___|___|

14.	When did you first meet [initials]?<br>**G1E14A, G1E14B, G1E14C, G1E14F**<br>**G1E14AM, G1E14BM, G1E14CM, G1E14FM**<br>**G1E14AY, G1E14BY, G1E14CY, G1E14FY**	[ ] month<br>[ ] year
15.	How old is [initials]?<br>**G1E15A, G1E15B, G1E15C, G1E15F**	[ ] years
16.	What is or was your relationship to [initials]?<br>**G1E16A, G1E16AT**<br>**G1E16B, G1E16BT**<br>**G1E16C, G1E16CT**<br>**G1E16F, G1E16FT**	1 = He is or was my main partner/boyfriend<br>2 = He is or was a regular casual sex partner<br>3 = He is or was a non-regular casual sex partner<br>4 = He is or was a sex work client<br>5 = Other (specify)
17.	Is this relationship with [initials] ongoing or has it ended? **G1E17A, G1E17B, G1E17C**	1 = Ongoing<br>2 = Ended
18.	When was the last time you saw [initials]?<br>**G1E18A**<br>**G1E18B**<br>**G1E18C**<br>**G1E18F**	1 = Today<br>2 = Yesterday<br>3 = Between 2 to 10 days ago<br>4 = Between 11 days to 1 month ago<br>5 = Between 1 month to 3 months ago<br>6 = Over 3 months ago

19.	Where does [initials] live?<br>**G1E19A**<br>**G1E19B**<br>**G1E19C**<br>**G1E19F**	1 = In your village<br>2 = In another village in Bushbuckridge<br>3 = In another area within Mpumalanga<br>4 = In another province in South Africa<br>5 = Outside of South Africa<br>.D = I don't know

20.	Do you currently live with [initials]? **G1E20A, G1E20B, G1E20C, G1E20F**	1 = Yes<br>0 = No --> **Skip to #23**
21.	For how long have you been living together?<br>**G1E21A, G1E21B, G1E21C, G1E21F**	1 = Less than 6 months<br>2 = Between 6 months and 1 year<br>3 = More than 1 year
22.	In the last month, how many nights did you spend together with [initials]?<br>**G1E22A, G1E22B, G1E22C**	0 = 0<br>1 = 1-2<br>2 = 3-4<br>3 = 5 or more
23.	When did you FIRST have sex with [initials]?<br>**G1E23A G1E23AM G1E23AY**<br>**G1E23B G1E23BM G1E23BY**<br>**G1E23C G1E23CM G1E23CY G1E23CT**	[ ] month [ ] year
24.	When did you LAST have sex with [initials]?<br>**G1E24A G1E24AM G1E24AY**<br>**G1E24B G1E24BM G1E24BY**<br>**G1E24C G1E24CM G1E24CY G1E24CT**	[ ] month [ ] year
25.	On average, since the beginning of your relationship, how often have you had sex with [initials]?<br>**G1E25A**<br>**G1E25B**<br>**G1E25C**	1 = Once a day or more<br>2 = 3 to 6 times a week<br>3 = Once or twice a week<br>4 = 2 or 3 times a month<br>5 = Once a month or less<br>6 = One time
26.	On average, how often would you say you have used condoms when you have sex with [initials]? Would you say …<br>*[read all]*<br>**G1E26A, G1E26B, G1E26C**	1 = Never, none of the time<br>2 = Rarely, once in a while<br>3 = Sometimes, about half of the time<br>4 = Frequently, but not all the time<br>5 = Always, every time
27.	Did you use a condom with [initials] the last time you had sex? **G1E27A, G1E27B, G1E27C**	1 = Yes<br>0 = No
28.	Have you ever been pregnant by [initials]?<br>**G1E28A, G1E28B, G1E28C**	1 = Yes<br>0 = No --→**Skip to #31**

29.	Do you have children with [initials]?<br>**G1E29A, G1E29B, G1E29C**	1 = Yes<br>0 = No
30.	Does [initials] have any children by other women?<br>**G1E30A, G1E30B, G1E30C**	1 = Yes<br>2 = No<br>.D = I don't know

31.	As far as you know, during the time that you and [initials] have had a sexual relationship, has [initials] had any other sexual partners, such as girlfriends, wives or sex workers?<br>**G1E31A, G1E31B, G1E31BT, G1E31C**	1 = Yes<br>0 = No--> **Skip to #34**<br>.D = I don't know --> **Skip to #34**

32.	Besides you, how many other partners does [initials] have?<br>**G1E32A, G1E32B, G1E32C, G1E32AT, G1E31BT**	[ ] partners<br>.D = Don't know
33.	During the time that you and [initials] have had a sexual relationship, have you had any other sexual partners? **G1E33A G1E33B G1E33C**	1 = Yes<br>0 = No
34.	Does [initials] have HIV?<br>**G1E34A, G1E34B, G1E34C**	1 = Yes<br>0 = No<br>.D = I don't know --> **Skip to #37**
35.	How do you know [initial]'s HIV status?<br><br>*Pick all that apply.*<br><br>0 = No<br>1 = Yes	He told me **G1E35AA, G1E35BA, G1E35CA**<br>We took an HIV test together **G1E35AB, G1E35BB, G1E35CB**<br>He showed me the results of a test he took alone **G1E35AC, G1E35BC, G1E35CC**<br>I saw his HIV medication **G1E35AD, G1E35BD, G1E35CD**<br>He looks sick **G1E35AE, G1E35BE, G1E35CE**<br>He looks healthy **G1E35AF, G1E35BF, G1E35CF**<br>He has TB **G1E35AG, G1E35BG, G1E35CG**<br>He has only ever had sex with me **G1E35AH, G1E35BH, G1E35CH**<br>Someone else told me **G1E35AI, G1E35BI, G1E35CI**<br>Other (specify) **G1E35AJ, G1E35AT; G1E35BJ, G1E35BT; G1E35CJ, G1E35CT**
36.	Is [initials] currently enrolled in school?<br>**G1E36A, G1E36B, G1E36C, G1E36F**	1 = Yes<br>0 = No
37.	Does [initials] go to your school now, or did he in the past?<br>**G1E37A, G1E37B, G1E37C, G1E37F**	1 = Yes<br>0 = No
38.	What is the highest level of education that [initials] has achieved?<br>**G1E38A, G1E38B, G1E38C, G1E38F**	1 = No school<br>2 = Some primary<br>3 = Completed primary<br>4 = Some high school<br>5 = Completed high school<br>6 = University or Technikon<br>.D = I don't know
39.	Has [initials] ever given you money? **G1E39A, G1E39B, G1E39C, G1E39F**	1 = Yes<br>0 = No --> **Skip to #45**

40.	About how often has [initials] given you money? **G1E40A, G1E40B, G1E40C, G1E40F**	1 = Just once<br>2 = A few times a month<br>3 = Once a month<br>4 = Once a week<br>5 = More than once a week
41.	When the last time [initials] was gave you money? **G1E41A, G1E41B, G1E41C, G1E41F**	1 = This month<br>2 = Not this month, but within the past 6 months<br>3 = Between 6 months to a year ago<br>4 = Over a year ago
42.	On average, how much money did [initials] give you each time? **G1E42A, G1E42B, G1E42C, G1E42F**	[ ] Rands
43.	Did you feel like you had to have sex with [initials] because they gave you money? **G1E43A, G1E43B, G1E43C**	1 = Yes<br>0 = No
45.	Has [initials] ever given you things, like groceries, clothes or airtime, that help you get by? **G1E44A, G1E44B, G1E44C, G1E44F**	1 = Yes<br>0 = No --> **Skip to #49**
46.	About how often has [initials] given you things? **G1E45A, G1E45B, G1E45C, G1E45F**	1 = Once<br>2 = A few times<br>3 = Often<br>4 = Always
47.	When the last time [initials] was gave you things? **G1E46A, G1E46B, G1E46C, G1E46F**	1 = This month<br>2 = Not this month, but within the past 6 months<br>3 = Between 6 months to a year ago<br>4 = Over a year ago
48.	What type of things has he given you?<br><br>*Pick all that apply.*<br><br>1 = Yes<br>0 = No	Airtime **G1E47FA, G1E47AA - G1E47CA**<br>Cell phone **G1E47FB , G1E47AB- G1E47CB**<br>Groceries **G1E47FC, G1E47AC- G1E47CC**<br>Clothes or shoes **G1E47FD, G1E47AD- G1E47CD**<br>Perfume or lotions **G1E47FE, G1E47AE- G1E47CE**<br>Make-up **G1E47FF, G1E47AF- G1E47CF**<br>Cool drink, sweets or chips **G1E47FG, G1E47AG- G1E47CG**<br>CDs, DVDs or videos **G1E47FH, G1E47AH- G1E47CH**<br>Alcohol or drugs **G1E47FI, G1E47AI- G1E47CI**<br>Flowers **G1E47FJ, G1E47AJ- G1E47CJ**<br>Other (specify) **G1E47FL, G1E47FT, G1E47AL- G1E47CL, G1E47AT- G1E47CT**

49.	Did you feel like you had to have sex with [initials] because they gave you things? **G1E48A- G1E48C**	1 = Yes<br>0 = No
50.	Have you ever talked with [initials] about condom use? **G1E49A- G1E49C, G1E49F**	1 = Yes<br>0 = No
51.	Have you ever talked with [initials] about preventing HIV? **G1E50F, G1E50A- G1E50C**	1 = Yes<br>0 = No
52.	Have you ever talked with [initials] about getting tested for HIV? **G1E51F, G1E51A- G1E51C**	1 = Yes<br>0 = No

<u>HEALTH & FERTILITY</u> -- MIT Kenya & RDS Zimbabwe

Interviewer script: *Now I would like to ask you some questions about your health. We will start by talking about your pregnancy history.*

1.	Have you ever been pregnant? **G1F1**	1 = Yes<br>0 = No --> **Skip to #10**
2.	What was your age at first pregnancy? **PRIM_C**	[ ] age
3.	Have you ever been pregnant when you did not want to be? **G1F4**	1 = Yes<br>0 = No
4.	Did you stop attending school because of your pregnancy and/or baby? **G1F5**	1 = Yes<br>0 = No --> **Skip to #7**
5.	How long did you stop attending school for? **G1F6**	1 = 1 month<br>2 = 2-3 months<br>3 = 4-5 months<br>4 = 6-12 months<br>5 = More than 12 months
6.	How many times have you been pregnant? In addition to babies carried to term, this may include miscarriages, babies that were stillborn, or pregnancies that were terminated. **TIMPREG**	[ ] times
7.	How many living children do you have, that you have given birth to? **G1F8**	[ ] children
8.	How many children that you have given birth to live with you? **G1F9**	[ ] children If 0 **skip to #10**
9.	Are you able to find child care for the children that live with you, when needed? **G1F10**	1 = Yes<br>0 = No
10.	Are you using birth control now? **G1F11**	1 = Yes<br>0 = No --> **Skip to #12**
11.	What birth control method are you currently using?<br><br>*Pick all that apply.* ***Skip to #13***<br><br>0 = No<br>1 = Yes	Birth control pills **BCMETH1**<br>Injectables: Depo Provera (3 monthly injection) **BCMETH2**<br>Injectables: NET-EN (known as Nuristerate, 2 monthly injection) **BCMETH3**<br>Male condoms **BCMETH4**<br>Female condoms **BCMETH5**<br>IUD/Mirena **BCMETH6**<br>Counting days (rhythm method; observation of menstrual cycle) **BCMETH7**<br>Traditional methods **BCMETH8**<br>Withdrawal **BCMETH9**

12.	Why are you not currently using birth control?<br><br>*Pick all that apply.*<br><br>0 = No<br>1 = Yes	You are not in a sexual relationship **G1F13A**<br>It is too expensive **G1F13B**<br>You do not know where to get it **G1F13C**<br>You are embarrassed to ask for it **G1F13D**<br>You do not know about or understand birth control **G1F13E**<br>You did not think about it **G1F13F**<br>You are worried about the side effects **G1F13G**<br>A healthcare worker refused to give you birth control **G1F13H**<br>You are opposed to birth control **G1F13I**<br>Other (specify) **G1F13J, G1F13JT**
13.	How important is it for you to keep from getting pregnant now?<br>**G1F14**	1 = Not important<br>2 = Somewhat important<br>3 = Very important
14.	Compared to the other things in your life, how much do you worry about getting pregnant?<br>**G1F15**	1 = Not at all<br>2 = Somewhat<br>3 = A lot
15.	What do you think are the chances that you will fall pregnant before you finish school? **G1F16**	1 = No chance<br>2 = Some chance<br>3 = A very high chance

Interviewer script: Now we will continue by asking questions about drugs and alcohol.

16.	How often do you drink alcohol (including wine, beer)?<br>**G1F17, G1F17T**	1 = Never --> **Skip to #19**<br>2 = Less than once a month<br>3 = Once a month<br>4 = 2-3 times a month<br>5 = Once a week<br>6 = 2-6 times a week<br>7 = Daily
17.	On a day that you have an alcoholic drink, how many standard drinks do you usually have? *A standard drink is a small glass of wine; a 330 ml can of regular beer, a tot of spirits, or a mixed drink.* **G1F18**	1 = 1 or 2 standard drinks<br>2 = 3 or 4 standard drinks<br>3 = 5 or 6 standard drinks<br>4 = More than 6 standard drinks
18.	Have you ever had sex while you were drunk on alcohol? --> *Skip if No to questions 2 and 3 in PG*<br>**G1F19**	1 = Yes<br>0 = No
19.	Have you ever used drugs? Drugs may include dagga, mandrax, tik, glue, or any substance you use to get high. **G1F20**	1 = Yes<br>0 = No --> END MODULE
20.	Have you ever had sex while you were high on drugs? --> *Skip if No to questions 2 and 3 in PG*<br>**G1F21**	1 = Yes<br>0 = No

<u>CONSUMPTION</u> -- from Malawi CCT

Interviewer script: *Now I would like to ask you questions about items you may have purchased for yourself or someone else during the past month.*

Coding Instructions: Ask all possible items in #1 first and then proceed to #2 for affirmative responses; same coding for #3 and #4.

		1. Over the past month, did you buy for yourself any […]?<br><br>1 = Yes<br>0 = No	2. Over the past month, how much money in rands did you spend on […] for yourself?	3. Over the past month, did you buy for anyone else any […]?<br><br>1 = Yes<br>0 = No	4. Over the past month, how much money in rands did you spend on […] for anyone else?
1.	Scented soap, skin creams or lotions	G1H1A	G1H2A	G1H3A	G1H4A
2.	Cell phone, airtime, or ringtones	G1H1B	G1H2B	G1H3B	G1H4B
3.	Shoes, clothing or underwear	G1H1C	G1H2C	G1H3C	G1H4C
4.	Make-up or cosmetics	G1H1D	G1H2D	G1H3D	G1H4D
5.	Hairdressing	G1H1E	G1H2E	G1H3E	G1H4E
6.	Chips, sweets, cool drinks	G1H1F	G1H2F	G1H3F	G1H4F
7.	Food/groceries (not cool drink/chips/sweets)	G1H1G	G1H2G	G1H3G	G1H4G
8.	Movies or music	G1H1H	G1H2H	G1H3H	G1H4H
9.	Beer or other alcoholic drinks	G1H1I	G1H2I	G1H3I	G1H4I
10.	Birth control and/or condoms	G1H1J	G1H2J	G1H3J	G1H4J
11.	Transport to work, school, or anywhere else	G1H1K	G1H2K	G1H3K	G1H4K
12.	School uniform, fees, books, or supplies	G1H1M	G1H2M	G1H3M	G1H4M

<u>SEXUAL RELATIONSHIP POWER SCALE</u> – South African Adaptation

Coding instructions: If ppt responded NO to #2 AND #3 on Partner Grid, skip SRPS

Interviewer script: *We would now like to ask you some questions about your relationship with your current or most recent boyfriend and for each we would like you to tell us how much you agree with each statement. Please tell us if you agree a lot, somewhat agree, or do not agree at all with the statement.*

1.	If I asked my partner to use a condom, he would beat or hit me. **G1L1**	1 = Agree a lot<br>2 = Somewhat agree<br>3 = Do not agree at all
2.	If I asked my partner to use a condom, he would get angry. **G1L2**	1 = Agree a lot<br>2 = Somewhat agree<br>3 = Do not agree at all
3.	My partner won't let me wear certain things. **G1L3**	1 = Agree a lot<br>2 = Somewhat agree<br>3 = Do not agree at all
4.	My partner has more to say than I do about important decisions that affect us. **G1L4**	1 = Agree a lot<br>2 = Somewhat agree<br>3 = Do not agree at all
5.	My partner tells me who I can spend time with. **G1L5**	1 = Agree a lot<br>2 = Somewhat agree<br>3 = Do not agree at all
6.	If I asked my partner to use a condom, he would think I am having sex with other people. **G1L6**	1 = Agree a lot<br>2 = Somewhat agree<br>3 = Do not agree at all
7.	I feel trapped or stuck in our relationship. **G1L7**	1 = Agree a lot<br>2 = Somewhat agree<br>3 = Do not agree at all
8.	My partner does what he wants, even if I don't want him to. **G1L8**	1 = Agree a lot<br>2 = Somewhat agree<br>3 = Do not agree at all
9.	When my partner and I disagree, he gets his way most of the time. **G1L9**	1 = Agree a lot<br>2 = Somewhat agree<br>3 = Do not agree at all
10.	My partner always wants to know where I am. **G1L10**	1 = Agree a lot<br>2 = Somewhat agree<br>3 = Do not agree at all
11.	My partner is having sex with someone else. **G1L11**	1 = Agree a lot<br>2 = Somewhat agree<br>3 = Do not agree at all
12.	Because my partner buys me things I want to please him. **G1L12**	1 = Agree a lot<br>2 = Somewhat agree<br>3 = Do not agree at all

GENDER EQUITABLE MEN'S SCALE

Interviewer script: *We would now like to ask you some questions about relationships between men and women. For each statement, please tell us if you agree a lot, somewhat agree, or do not agree at all with the statement.*

Violence Domain		
1.	There are times when a woman deserves to be beaten. **G1L13**	1 = Agree a lot<br>2 = Somewhat agree<br>3 = Do not agree at all
2.	A woman should tolerate violence to keep her family together. **G1L14**	1 = Agree a lot<br>2 = Somewhat agree<br>3 = Do not agree at all
3.	It is alright for a man to beat his wife if she is unfaithful. **G1L15**	1 = Agree a lot<br>2 = Somewhat agree<br>3 = Do not agree at all
4.	A man can hit his wife if she won't have sex with him. **G1L16**	1 = Agree a lot<br>2 = Somewhat agree<br>3 = Do not agree at all
5.	If someone insults a man he should defend his reputation with force if he has to. **G1L17**	1 = Agree a lot<br>2 = Somewhat agree<br>3 = Do not agree at all
6.	A man using violence against his wife is a private matter that shouldn't be discussed outside the couple. **G1L18**	1 = Agree a lot<br>2 = Somewhat agree<br>3 = Do not agree at all
Sexual Relationships Domain		
7.	It is the man who decides what type of sex to have. **G1L19**	1 = Agree a lot<br>2 = Somewhat agree<br>3 = Do not agree at all
8.	Men are always ready to have sex. **G1L20**	1 = Agree a lot<br>2 = Somewhat agree<br>3 = Do not agree at all
9.	Men need sex more than women do. **G1L21**	1 = Agree a lot<br>2 = Somewhat agree<br>3 = Do not agree at all
10.	A man needs other women even if things with his wife/partner are fine. **G1L22**	1 = Agree a lot<br>2 = Somewhat agree<br>3 = Do not agree at all
11.	You don't talk about sex, you just do it. **G1L23**	1 = Agree a lot<br>2 = Somewhat agree<br>3 = Do not agree at all

12.	It disgusts me when I see a man acting like a woman. **G1L24**	1 = Agree a lot<br>2 = Somewhat agree<br>3 = Do not agree at all
13.	A woman should not initiate sex. **G1L25**	1 = Agree a lot<br>2 = Somewhat agree<br>3 = Do not agree at all
14.	A woman who has sex before she marries does not deserve respect. **G1L26**	1 = Agree a lot<br>2 = Somewhat agree<br>3 = Do not agree at all
Reproductive Health and Disease Prevention Domain		
15.	Women who carry condoms on them are easy. **G1L27**	1 = Agree a lot<br>2 = Somewhat agree<br>3 = Do not agree at all
16.	Men should be outraged if their wives/partners ask them to use a condom. **G1L28**	1 = Agree a lot<br>2 = Somewhat agree<br>3 = Do not agree at all
17.	It is a woman's responsibility to avoid getting pregnant. **G1L29**	1 = Agree a lot<br>2 = Somewhat agree<br>3 = Do not agree at all
18.	Only when a woman has a child is she a real woman. **G1L30**	1 = Agree a lot<br>2 = Somewhat agree<br>3 = Do not agree at all
19.	A real man produces a male child**.** **G1L31**	1 = Agree a lot<br>2 = Somewhat agree<br>3 = Do not agree at all
Domestic Chores & Daily Life		
20.	Changing diapers, giving a bath, and feeding kids is the mother's responsibility. **G1L32**	1 = Agree a lot<br>2 = Somewhat agree<br>3 = Do not agree at all
21.	A woman's role is taking care of her home and family. **G1L33**	1 = Agree a lot<br>2 = Somewhat agree<br>3 = Do not agree at all
22.	The husband should decide to buy the major household items. **G1L34**	1 = Agree a lot<br>2 = Somewhat agree<br>3 = Do not agree at all
23.	A man should have the final word about decisions in his home. **G1L35**	1 = Agree a lot<br>2 = Somewhat agree<br>3 = Do not agree at all
24.	A woman should obey her husband in all things. **G1L36**	1 = Agree a lot<br>2 = Somewhat agree<br>3 = Do not agree at all

INTIMATE PARTNER VIOLENCE-- ***from WHO***

Interviewer script: *We would like to ask you some questions about violence within relationships. We will ask you about ways a partner may have hurt you. When we say "partner," we mean your current boyfriend or partner or any other partner in your past. We'd like to know if any of the situations we describe have ever happened to you in your life.*

This section will ask you some questions related to physical and sexual violence and abuse. ***Because you are under 18 years old, if you decide to answer these questions and you report having experienced physical or sexual violence or abuse we will be obliged to find help for you per South African Department of Health guidelines*** *. This includes having to report the physical or sexual violence to a social worker because you are still a minor (less than18 years).*

	A) Has this ever happened?	B) Has this happened in the past 12 months?
1. Has a partner ever slapped you or thrown something at you that could hurt you?	1 = Yes<br>0 = No --> **Skip to next question**<br>REF --> **Skip to next question**<br>**G1D1A**	1 = Yes<br>0 = No<br>**G1D1B**
2. Has a partner ever pushed you or shoved you?	1 = Yes<br>0 = No --> **Skip to next question**<br>REF --> **Skip to next question**<br>**G1D2A**	1 = Yes<br>0 = No<br>**G1D2B**
3. Has a partner ever hit you with their fist or with something else that could hurt?	1 = Yes<br>0 = No --> **Skip to next question**<br>REF --> **Skip to next question**<br>**G1D3A**	1 = Yes<br>0 = No<br>**G1D3B**
4. Has a partner ever kicked you, dragged you, or beaten you up?	1 = Yes<br>0 = No --> **Skip to next question**<br>REF --> **Skip to next question**<br>**G1D4A**	1 = Yes<br>0 = No<br>**G1D4B**
5. Has a partner ever choked or burnt you on purpose?	1 = Yes<br>0 = No --> **Skip to next question**<br>REF --> **Skip to next question**<br>**G1D5A**	1 = Yes<br>0 = No<br>**G1D5B**
6. Has a partner ever threatened to use or actually used a gun, knife or other weapon against you?	1 = Yes<br>0 = No --> **Skip to next question**<br>REF --> **Skip to next question**<br>**G1D6A**	1 = Yes<br>0 = No<br>**G1D6B**

Interviewer script: We would like to ask you some sensitive questions about past sexual experiences. These questions are not meant to make you feel uncomfortable. We need to ask about these things so that we can get as much information as possible about the experiences of young people today. Remember that all of your answers are confidential. If there is a question that you really do not feel comfortable answering, please let us know and we can skip that question. We really appreciate you sharing as much information with us as possible.

	A) Has this ever happened?	B) Has this happened in the past 12 months?	C) Was this done by a boyfriend or partner?	D) Was this done by a family member?	E) Was this done by someone else outside your family (not including a boyfriend or partner)?
7. Has anyone ever physically forced you to have sexual intercourse when you did not want to?	1 = Yes<br>0 = No --> **Skip to next question**<br>**G1D7A**	1 = Yes<br>0 = No<br>**G1D7B**	1 = Yes<br>0 = No<br>**G1D7C**	1 = Yes<br>0 = No<br>**G1D7D**	1 = Yes<br>0 = No<br>**G1D7E**
8. Have you ever had sexual intercourse that you did not want because you were afraid of what the other person might do?	1 = Yes<br>0 = No --> **Skip to next question**<br>**G1D8A**	1 = Yes<br>0 = No<br>**G1D8B**	1 = Yes<br>0 = No<br>**G1D8C**	1 = Yes<br>0 = No<br>**G1D8D**	1 = Yes<br>0 = No<br>**G1D8E**

<u>HIV KNOWLEDGE</u> -- NBCS Zimbabwe Baseline Questionnaire and RADARP

Interviewer script. Now we would like to ask you some questions about HIV. For each statement, please tell me if it is true or false.

1.	A person who looks strong and healthy can have HIV.<br>**G1I1**	1 = True<br>2 = False<br>.D = I don't know
2.	You can get HIV if you share utensils with someone who is infected.<br>**G1I2**	1 = True<br>2 = False<br>.D = I don't know
3.	If a mosquito bites you it can infect you with HIV.<br>**G1I3**	1 = True<br>2 = False<br>.D = I don't know
4.	You cannot get HIV from kissing a person who is HIV-positive.<br>**G1I4**	1 = True<br>2 = False<br>.D = I don't know
5.	A woman who is pregnant can do nothing to prevent her baby from being born with HIV. **G1I5**	1 = True<br>2 = False<br>.D = I don't know
6.	Treatment is available for HIV/AIDS.<br>**G1I6**	1 = True<br>2 = False<br>.D = I don't know

Interviewer script. *Now we would like to ask you some questions about HIV and your life.*

7.	What have you done to reduce your chances of getting HIV? *Pick all that apply*<br><br>1 = Yes<br>0 = No	Never had sex **G1I7A**<br>Stopped having sex or abstained from sex **G1I7B**<br>Used condoms every time you had sex **G1I7C**<br>Changed the way you select who you have sex with **G1I7D**<br>Reduced your number of sex partners **G1I7E**<br>Had sex with only one partner **G1I7F**<br>Had an HIV test **G1I7G**<br>Chosen only healthy-looking people as your sexual partners **G1I7H**<br>None of these **G1I7I**
8.	What do you think are the chances that you will get HIV/AIDS in your lifetime?<br>**G1I8**	1 = No chance<br>2 = Some chance<br>3 = A very high chance
9.	Before this study, have you ever tested for HIV? **G1I9**	1 = Yes<br>0 = No

FAMILY

Interviewer script: Now we would like to ask you some questions about your relationship with the people in your life that care for you. Your primary caregiver is the adult or adults most responsible for taking care of you.

1.	Which adult is most involved in your schooling? **G1J1**<br><br>***Coding: List the person chosen for #1 in question #2-7***	1 = Mother<br>2 = Father<br>3 = Older brother<br>4 = Older Sister<br>5 = Aunt<br>6 = Uncle<br>7 = Grandparent<br>8 = Cousin<br>9 = Other adult who is not a blood relative
2.	How often does [ ] check to see that you've done your school work?<br>**G1J2**	1 = Never<br>2 = Sometimes<br>3 = Always
3.	How often does [ ] help you with your school work?<br>**G1J3**	1 = Never<br>2 = Sometimes<br>3 = Always
4.	During the most recent school year, how often did you discuss things you studied in class with [ ]?<br>**G1J4**	1 = Never<br>2 = Sometimes<br>3 = Always<br>4 = N/A (not in school)
5.	During the most recent school year, how often did you discuss your marks with [ ]?<br>**G1J5**	1 = Never<br>2 = Sometimes<br>3 = Always<br>4 = N/A (not in school)
6.	How far in school does [ ] want you to go?<br>**G1J6**	1 = Finish the current year and no more<br>2 = Pass matric<br>3 = University or technikon
7.	If you dropped out of school, how disappointed would [ ] be? **G1J7**<br>If you have previously dropped out of school, tell us how disappointed they were at that time.	1 = Very much<br>2 = Somewhat<br>3 = Not at all
8.	Which adult is most involved in other areas of life (NOT including schooling)? **G1J8**<br><br>***Coding: List the person chosen for #8 in question #9-16***	1 = Mother<br>2 = Father<br>3 = Older brother<br>4 = Older Sister<br>5 = Aunt<br>6 = Uncle<br>7 = Grandparent<br>8 = Cousin<br>9 = Other adult who is not a blood relative
9.	How often does [ ] try to find out about who your friends are?<br>**G1J9**	1 = Never<br>2 = Sometimes<br>3 = Always
10.	How often does [ ] try to find out about how you spend your free time? **G1J10**	1 = Never<br>2 = Sometimes<br>3 = Always

11.	How often does [ ] try to find out about where you go after school?<br>**G1J11**	1 = Never<br>2 = Sometimes<br>3 = Always
12.	How often does [ ] try to find out about how you spend your money? **G1J12**	1 = Never<br>2 = Sometimes<br>3 = Always
13.	How much do you feel that [ ] cares about you?<br>**G1J13**	1 = Very much<br>2 = Somewhat<br>3 = Not at all
14.	How close do you feel to [ ]?<br>**G1J14**	1 = Very close<br>2 = Somewhat close<br>3 = Not at all close
15.	How free/open do you feel to discuss issues of sex and sexuality with [ ]? **G1J15**	1 = Very much<br>2 = Somewhat<br>3 = Not at all
16.	How free/open do you feel to discuss issues of HIV prevention with [ ]? **G1J16**	1 = Very much<br>2 = Somewhat<br>3 = Not at all

Interviewer script: *Now we will ask about places that you may visit and clubs or associations to which you might belong.*

17.	How many times in the past six months have you been to:	A funeral? [ ] **G1J17A**<br>A wedding? [] **G1J17B**<br>A tavern? [] **G1J17C**<br>A place where people dance? [] **G1J17D**<br>A shop? [] **G1J17E**<br>A church? [] **G1J17F**<br>A choir event? [ ] **G1J17G**<br>A sport event or game? [] **G1J17H**<br>Another school-related extracurricular activity? [] **G1J17I**<br>A political meeting? [] **G1J17J**<br>A stokvel [] **G1J17K**<br>An HIV awareness or testing event [ ] **G1J17L**
18.	If you are a member of a group or club, please select which types of groups to which you belong.<br><br>*Pick all that apply.*<br><br>1 = Yes<br>0 = No	Student group **G1J18A**<br>Health group **G1J18B**<br>HIV/AIDS group **G1J18C**<br>Finance/loan group **G1J18D**<br>Church or religious group **G1J18E**<br>Sport group **G1J18F**<br>Dance group **G1J18G**

<u>EMPLOYMENT & FINANCES</u> -- From Malawi CCT and Project CONNECT

Interviewer script: *For this next section, I will ask you questions about activity, jobs and finances.*

1.	I want you to think of a typical school day **now** in your life, when schools are in session. Can you choose the top 3 activities you spend your time doing in a typical day?<br><br>1 = Yes<br>0 = No	Doing homework **G1G1A_0**<br>Housework/cleaning/cooking **G1G1B_0**<br>Childcare/watching children **G1G1C_0**<br>Working in the garden/farm pilot **G1G1D_0**<br>Working family store/business **G1G1E_0**<br>Working outside home for pay **G1G1F_0**<br>Spending time with friends **G1G1G_0**<br>Spending time with boyfriend **G1G1H_0**<br>Playing sports **G1G1I_0**<br>Church-related activities **G1G1J_0**<br>Extracurricular activities **G1G1K_0**<br>School **G1G1L_0**
2.	Did you do any work for pay or family gain, including payment in kind such as food or housing? **G1G2**<br><br>When I ask about work, I am asking about anything that you have done for money, for payment in kind (such as food) or for family gain. Please tell me even if the work was not a proper job.	1 = Yes<br>0 = No --> **Skip to #8**
3.	During the past 12 months, of the work you did for pay or family gain, what kind of work did you spend most of your time on?<br><br>*Pick all that apply.*<br><br>1 = Yes<br>0 = No	Domestic worker **G1G3A**<br>Child care **G1G3B**<br>Farm worker **G1G3C**<br>Mining **G1G3D**<br>Tourism/game parks **G1G3E**<br>Working in a shop **G1G3F**<br>Informal selling **G1G3G**<br>Sex work **G1G3H**<br>Tavern or restaurant **G1G3I**<br>Transport **G1G3J**<br>Factory worker **G1G3K**<br>Clerical and office work **G1G3L**<br>Small business assistant **G1G3M**<br>Sewing, hairdressing, baking or brewing **G1G3N**<br>Other (specify) **G1G3O, G1G3T**
4.	How many hours in an average week do you spend doing this work? **G1G4**	[ ] Hours

5.	Think about all the work that you have done in the past 3 months to earn money or goods. How much do you estimate that you have earned in the past 3 months? **G1G5**	[ ] Rands
6.	What is the main reason you work? *Pick one.* **G1G6**	1 = I want money for myself<br>2 = To support my family<br>3 = Something to do<br>4 = A way to meet friends

7.	How often in the past 12 months has your job interfered with you attending school or completing your school work? **G1G7**	1 = Never<br>2 = A few times<br>3 = Always
8.	During the past 12 months, where did you get most of your money?<br>*Pick only one response.*<br>**G1G8, G1G8T**	1 = Job<br>2 = Family<br>3 = Friends<br>4 = Boyfriend or partner<br>5 = Swa Koteka<br>6 = Grants (child support, disability, etc.)<br>7 = Sex Work<br>8 = Selling drugs<br>9 = Begging/Shoplifting/Etc.<br>10 = Didn't have any money<br>11 = Other (specify)
9.	How often in the past 12 months have you had your own money to spend however you like?<br>**G1G9**	1 = Never --> If never, **skip to #11**<br>2 = A few times<br>3 = Always
10.	During the past 12 months, about how much money have you usually had each month to spend however you like? **G1G10**	[ ] Rands
11.	In the past 12 months, have you been worried about having enough food for you or your family?<br>**G1G11**	1 = Yes<br>0 = No
12.	In the past 12 months, how often have you had to borrow money from a friend or someone outside of your household to get by?<br>**G1G12**<br>*Pick one.*	1 = More than 5 times<br>2 = 3-5 times<br>3 = 1-2 times<br>4 = Never
13.	Do you currently have any savings for the future, such as cash or assets?<br>**G1G13**	1 = Yes<br>0 = No --> **Skip to #15**
14.	What is the approximate amount of money that you currently have in savings? **G1G14**	[ ] Rands
15.	Do you currently have a bank or post office account?<br>**G1G15**	1 = Yes<br>0 = No

SELF-EFFICACY

Interviewer script: *Now we will read some statements about sex and condoms. For each statement, please tell us if you agree a lot, somewhat agree, or do not agree at all with the statement.*

1.	I can ask a new partner to use condoms.<br>**G1M1**	1 = Agree a lot<br>2 = Somewhat agree<br>3 = Do not agree at all
2.	I can ask a partner I haven't been using condoms with to start using them.<br>**G1M2**	1 = Agree a lot<br>2 = Somewhat agree<br>3 = Do not agree at all
3.	I can refuse sex when I don't have a condom available.<br>**G1M3**	1 = Agree a lot<br>2 = Somewhat agree<br>3 = Do not agree at all
4.	I can get a partner to use condoms, even if I'm drunk or high.<br>**G1M4**	1 = Agree a lot<br>2 = Somewhat agree<br>3 = Do not agree at all
5.	I can get every partner to use male condoms, even if they don't want to.<br>**G1M5**	1 = Agree a lot<br>2 = Somewhat agree<br>3 = Do not agree at all
6.	I can find condoms if I want to use them.<br>**G1M6**	1 = Agree a lot<br>2 = Somewhat agree<br>3 = Do not agree at all

REVISED CHILDREN'S MANIFEST ANXIETY SCALE

Interviewer Script: *Many kids and teenagers feel nervous or anxious at times. Please say which of these is true for you.*

1.	I worry a lot of the time **G1O1**	1 = Yes<br>0 = No
2.	I worry about what my carers will say to me. **G1O2**	1 = Yes<br>0 = No
3.	I feel that others do not like the way I do things. **G1O3**	1 = Yes<br>0 = No
4.	It is hard for me to get to sleep at night. **G1O4, G1O4T**	1 = Yes<br>0 = No
5.	I worry about what other people think about me **G1O5**	1 = Yes<br>0 = No
6.	I feel alone even when there are people with me. **G1O6**	1 = Yes<br>0 = No
7.	I worry about what is going to happen. **G1O7**	1 = Yes<br>0 = No
8.	Other children/teenagers are happier than I. **G1O8**	1 = Yes<br>0 = No
9.	I have bad dreams. **G1O9**	1 = Yes<br>0 = No
10.	I wake up scared some of the time. **G1O10**	1 = Yes<br>0 = No
11.	I worry when I go to bed at night. **G1O11, G1O11T**	1 = Yes<br>0 = No
12.	I am nervous. **G1O12**	1 = Yes<br>0 = No
13.	A lot of people are against me. **G1O13**	1 = Yes<br>0 = No
14.	I often worry about something bad happening to me. **G1O14**	1 = Yes<br>0 = No

CHILDREN'S DEPRESSION INDEX

This part of the questionnaire looks at sadness and other difficulties which many people experience at some point in their lives. This questionnaire is arranged in groups of 3 statements. Please listen to each group carefully. Then pick out ONLY ONE statement from each group which best describes the way you have been feeling during the past 2 weeks.

1.	1 = I am sad once in a while **G1O15**<br>2 = I am sad many times<br>3 = I am sad all the time
2.	1 = Nothing will ever work out for me **G1O16**<br>2 = I am not sure if things will work out for me<br>3 = Things will work out for me ok
3.	1 = I do most things ok **G1O17**<br>2 = I do many things wrong<br>3 = I do everything wrong
4.	1 = I hate myself **G1O18**<br>2 = I do not like myself<br>3 = I like myself
5.	1 = I feel like crying every day **G1O19**<br>2 = I feel like crying many days<br>3 = I feel like crying once in awhile
6.	1 = Things bother me all the time **G1O20**<br>2 = Things bother me many times<br>3 = Things bother me once in awhile
7.	1 = I look ok **G1O21**<br>2 = There are some bad things about my looks<br>3 = I look ugly
8.	1 = I do not feel alone **G1O22**<br>2 = I feel alone many times<br>3 = I feel alone all the time
9.	1 = I have plenty of friends **G1O23**<br>2 = I have some friends but I wish I had more<br>3 = I don't have any friends
10.	1 = Nobody really loves me **G1O24**<br>2 = I am not sure if anybody loves me<br>3 = I am sure that somebody loves me

HOPE

Interviewer Script: *Now we would like to ask you some questions about your future and your hope for success. Please tell me how much you agree with the following statements about yourself on a scale of one to four, where 1= I totally disagree, 2= I disagree, 3= I agree, and 4= I totally agree.*

1.	It is easy for me to set goals. **G1P1**	1 = I totally disagree<br>2 = I disagree<br>3 = I agree<br>4 = I totally agree
2.	I believe that good things happen to me. **G1P2**	1 = I totally disagree<br>2 = I disagree<br>3 = I agree<br>4 = I totally agree
3.	I do not worry too much about problems now because I believe my life will be better in the future. **G1P3**	1 = I totally disagree<br>2 = I disagree<br>3 = I agree<br>4 = I totally agree
4.	My parents/guardians support me to achieve my goals. **G1P4**	1 = I totally disagree<br>2 = I disagree<br>3 = I agree<br>4 = I totally agree
5.	I know that the future is under my control even if things go wrong. **G1P5**	1 = I totally disagree<br>2 = I disagree<br>3 = I agree<br>4 = I totally agree
6.	I feel comfortable asking others for help when I need it to reach a goal. **G1P6**	1 = I totally disagree<br>2 = I disagree<br>3 = I agree<br>4 = I totally agree
7.	Even when I fail, I keep trying because I know it will be better next time. **G1P7**	1 = I totally disagree<br>2 = I disagree<br>3 = I agree<br>4 = I totally agree
8.	I enjoy thinking about how I am going to achieve what I want in my future. **G1P8**	1 = I totally disagree<br>2 = I disagree<br>3 = I agree<br>4 = I totally agree
9.	There is nothing that can get in my way of having a good future. **G1P9**	1 = I totally disagree<br>2 = I disagree<br>3 = I agree<br>4 = I totally agree
10.	I trust that I will be able to do everything that I want to do in my future. **G1P10**	1 = I totally disagree<br>2 = I disagree<br>3 = I agree<br>4 = I totally agree
11.	I will be successful because I know other people like me whom have been successful. **G1P11**	1 = I totally disagree<br>2 = I disagree<br>3 = I agree<br>4 = I totally agree
12.	I have more confidence in my future success than others my age. **G1P12**	1 = I totally disagree<br>2 = I disagree<br>3 = I agree<br>4 = I totally agree

13.	I am the kind of person who makes plans for how to reach my dreams. **G1P13**	1 = I totally disagree<br>2 = I disagree<br>3 = I agree<br>4 = I totally agree
14.	I believe that the things I am doing now are preparing me for what I want in the future. **G1P14**	1 = I totally disagree<br>2 = I disagree<br>3 = I agree<br>4 = I totally agree
15.	My friends and I share the dream to have a successful future. **G1P15**	1 = I totally disagree<br>2 = I disagree<br>3 = I agree<br>4 = I totally agree
16.	I can achieve my dreams if I focus on it. **G1P16**	1 = I totally disagree<br>2 = I disagree<br>3 = I agree<br>4 = I totally agree
17.	I trust that I will achieve the goals that I set for myself. **G1P17**	1 = I totally disagree<br>2 = I disagree<br>3 = I agree<br>4 = I totally agree
18.	The important people in my life tell me that I will have a successful life. **G1P18**	1 = I totally disagree<br>2 = I disagree<br>3 = I agree<br>4 = I totally agree
19.	I know that my life will be better in the future. **G1P19**	1 = I totally disagree<br>2 = I disagree<br>3 = I agree<br>4 = I totally agree
20.	It is easy for me to reach my goals. **G1P20**	1 = I totally disagree<br>2 = I disagree<br>3 = I agree<br>4 = I totally agree
21.	I believe that I will be successful even when there are difficulties in my life now. **G1P21**	1 = I totally disagree<br>2 = I disagree<br>3 = I agree<br>4 = I totally agree
22.	There are people who can help me when I need guidance to achieve something important to me. **G1P22**	1 = I totally disagree<br>2 = I disagree<br>3 = I agree<br>4 = I totally agree
23.	I have faith that I will be successful. **G1P23**	1 = I totally disagree<br>2 = I disagree<br>3 = I agree<br>4 = I totally agree
24.	I am careful about what I am doing now because it could affect my plans for the future. **G1P24**	1 = I totally disagree<br>2 = I disagree<br>3 = I agree<br>4 = I totally agree
25.	When I feel discouraged my religious beliefs help me the most. **G1P25**	1 = I totally disagree<br>2 = I disagree<br>3 = I agree<br>4 = I totally agree

FRIEND GRID

Interviewer script: *Now we want to ask you about five of your friends. We will ask you for initials for each friend. The initials you choose are only to help you remember which friend you are talking about as you answer questions, not to identify your friends. Over the following few minutes we are going to ask you some questions about each of these friends in turn. We will ask for information such as how long you have known each other, where your friend lives, and then something about what you talk about and what you do together. Please remember, we keep all information completely confidential.*

**Coding Instruction: Before proceeding, collect initials of all friends given by the participant. Only proceed to following questions after initials have been collected. When asking questions, please use the initials of the friend concerned.**		
1.	What are the initials of your friend?<br>**G1K1A – G1K1E G1K1CT**	[ ]
**Coding instruction: Identify relationships between all friends before proceeding**		
2.	What Is the relationship between Friend [#] and Friend [#, #, etc.]?<br>**G1K2A1 – G1K2A4, G1K2AT, G1K2A4T**<br>**G1K2B2 – G1K2B4,**<br>**G1K2B2T - G1K2B4T**<br>**G1K2C3, G1K2C4, G1K2C3T**<br>**G1K2D4**	0 = Never Met<br>1 = Friend<br>2 = Close friends<br>3 = Know each other but not friends<br>4 = Related by blood<br>5 = Related by marriage<br>6 = In a romantic/sexual relationship<br>7 = Other, specify
**Coding instructions: Record friend grid details for 5 friends total.**		
3.	What is this friend's gender? **G1K3A – G1K3E**	1 = Male<br>2 = Female
4.	What is this friend's home village?<br>**G1K4A – G1K4E, G1K4AT – G1K4ET**	[Village Codes]
5.	What is this friend's age? **G1K5A – G1K5E**	[ ] years
6.	Is this friend currently enrolled in school?<br>**G1K6A – G1K6E**<br><br>*If 'No' or 'Don't know' skip to #10*	1 = Yes<br>2 = No<br>.D = Don't know
7.	Is this friend currently enrolled in primary or secondary school?<br>**G1K7A – G1K7E**<br>*If Primary skip to #9*	1 = Primary<br>2 = Secondary<br>4 = University<br>.D = Don't know
8.	If secondary school, what is the most recent school this friend is/was enrolled in?<br>**G1K8A – G1K8E, G1K8AT – G1K8ET**	[school codes]
9.	If primary school, what is the most recent school this friend is/was enrolled in?<br>**G1K9A – G1K9E G1K9AT – G1K9ET**	[school codes]
10.	What was the main activity of this friend during last year? **G1K10A – G1K10E**	1 = Student<br>2 = Unemployed looking for work<br>3 = Unemployed doing housework, childcare, etc.<br>4 = Employed part-time or occasionally<br>5 = Employed full-time or regularly<br>6 = Does not work because sick, disabled, pregnant<br>7 = Singing in a school choir<br>8 = Marathon sports

Characteristics of friendship		
11.	How long have you known this friend?<br>**G1K11A – G1K11E**	1 = Less than one month<br>2 = 1-6 months<br>3 = 7-12 months<br>4 = 1-2 years<br>5 = 3-5 years<br>6 = More than 5 years, but not my whole life<br>7 = My whole life
12.	Is this friend a blood relative of yours?<br>**G1K12A – G1K12E**<br>*If No skip to #14*	1 = Yes<br>0 = No
13.	Describe relationship. This friend is my:<br><br>*Skip to #15*<br><br>**G1K13A – G1K13E**	1 = Brother<br>2 = Sister<br>3 = Half brother<br>4 = Half sister<br>5 = Mother<br>6 = Father<br>7 = Son<br>8 = Daughter<br>9 = Aunt<br>10 = Uncle<br>11 = Cousin (Mother's sisters/brother child)<br>12 = Cousin (Father's sisters/brothers child)<br>13 = Other blood relative<br>14 = Sister-in-law
14.	Is this friend your boyfriend or sexual partner?<br>**G1K14A – G1K14E**<br>*Skip if answered 'female' to Q3*	1 = Yes<br>0 = No
15.	How often do you see this friend?<br>**G1K15A – G1K15E**<br>**G1K15DT, G1K15ET**	1 = Everyday<br>2 = Most days each week<br>3 = A few times a week<br>4 = Less than once a week<br>5 = School holidays<br>6 = Every month<br>7 = Last year<br>8 = Once a year<br>9 = One or twice a month<br>10 = On vacation<br>90 = Other, specify
16.	Where do you usually spend time with this friend?<br>*Mark all that apply*<br><br>1 = Yes<br>0 = No	At school **G1K16AA – G1K16EA**<br>At church **G1K16AB – G1K16EB**<br>At my home **G1K16AC – G1K16EC**<br>At their home **G1K16AD – G1K16ED**<br>At football games/sporting events **G1K16AE – G1K16EE**<br>At meetings of a club/society/project **G1K16AF – G1K16EF**<br>At a tavern **G1K16AG – G1K16EG**<br>Around my village **G1K16AH – G1K16EH**<br>Around my friend's village **G1K16AI – G1K16EI**<br>Somewhere else outside of my community **G1K16AJ – G1K16EJ**<br>Other (specify) **G1K16AK – G1K16EK, G1K16AT – G1K16ET, G1K16CKT**

17.	Has this person ever introduced you to someone who later became a boyfriend/sexual partner? **G1K17A – G1K17E**	1 = Yes<br>0 = No
18.	Have you ever talked about your own sexual experiences with this friend? **G1K18A – G1K18E**	1 = Yes<br>0 = No
19.	Have you ever talked with this friend about whether you use condoms with your boyfriend/sexual partner? **G1K19A – G1K19E**	1 = Yes<br>0 = No
20.	Have you ever talked about HIV with this friend? **G1K20A – G1K20E**	1 = Yes<br>0 = No
21.	How would you describe the relationship between this friend and your current/most recent boyfriend/sexual partner **G1K21A – G1K21E**	1 = Same person<br>2 = Never met<br>3 = Know each other but not friends<br>4 = Related by blood<br>5 = Related by marriage<br>6 = Friend<br>7 = Close friends<br>8 = Never had boyfriend/sexual partner<br>9 = No boyfriend currently
22.	What is this friend's marital status? **G1K22A – G1K22E**<br>*If 'Married' skip to #24*	0 = Never married<br>1 = Married (legal or traditional)<br>2 = Separated or divorced<br>3 = Widowed
23.	Does this friend have a boy/girlfriend or regular sexual partner now? **G1K23A – G1K23E**	1 = Yes<br>2 = No<br>.D = Don't know
24.	Has this friend ever been pregnant? **G1K24A – G1K24E**<br>*Skip if answered 'male' in Q3*	1 = Yes<br>2 = No<br>.D = Don't know
25.	Do you think this friend has ever had sex? **G1K25A – G1K25E**	1 = Yes<br>0 = No
26.	Do you think this friend could refuse to have sex if their partner would not use a condom? **G1K26A – G1K26E**	1 = Yes<br>0 = No
27.	How often do you think this friend uses a condom with their main sexual partner? **G1K27A – G1K27E**<br>*Skip if answered 'no' to Q25*	1 = Never, none of the time<br>2 = Rarely, once in a while<br>3 = Sometimes, about half the time<br>4 = Frequently, but not all the time<br>5 = Always, every time
28.	Has this friend ever been tested for HIV? **G1K28A – G1K28E**	1 = Yes<br>0 = No<br>.D = don't know
29.	How many close friends do you have? **G1K29**	[ ] friends
30.	Thinking about people you discuss important matters with. Does this include any of the following?<br>*Mark all that apply*	Close friends **G1K30A**<br>Caregivers **G1K30B**<br>Other family members **G1K30C**<br>Teachers **G1K30D**

	1 = Yes<br>0 = No	Priest/Pastors **G1K30E**<br>Health professionals **G1K30F**<br>Other (specify) **G1K30G, G1K30T**

LIST OF CODES

**NOTE: (added) indicates not part of original answer options**	
**Village Codes**	
1	Agincourt
2	Croquet Lawn B
3	Croquet Lawn
4	Cunningmore A
5	Cunningmore B
6	Huntington
7	Ireagh A
8	Ireagh B
9	Justicia A or Justicia B
10	Kildaire A
11	Kildare B or Kildare C
12	Lillydale A or Rholane
13	Lillydale B
14	Newington B
15	Newington C
16	Somerset A or Somerset B
17	Xanthia
18	Khaya Lami
19	Dumphries A
20	Dumphries B
21	Dumphries C
22	Ireagh C
23	Somerset C
24	Belfast
25	Cork
26	Mkhuhlu
27	Khanimanba (added)
28	Johannesburg (added)
29	Giyani (Lipompo) (added)
30	Khanyisa (added)
31	Khayimani (added)
32	Makhosana (added)
33	Acornhoek (added)
34	Secunda (added)
35	Oakley (added)
36	Alexandra (added)
37	Merry Pebble Stream
38	Hazyview (added)
39	Rolle (added)
40	Phalaborwa (added)
41	Thulamahashe (added)
42	Ludlow (added)
43	Thembisa (added)
44	Dwarsloop, Dwarsloop B (added)
45	Khumani (added)
47	Shabalala (added)
48	Pretoria (added)
49	Ngodini (added)
50	Hokwe (added)
51	Hluvukani (added)
52	Gauteng (added)
53	Tekemahala (added)
54	Soweto (added)
55	Springs (added)
56	Mambhumbhu (added)
57	Slivia (added)
58	Rustinburg (added)
59	Witbank (added)
60	Xikontirini (added)
61	Humulani (added)
62	Vuyelani (added)
63	Tsuvulani (added)
64	Northwest (added)
65	Manukusa (added)
66	Makaringe (added)
67	White River (added)
68	Marite (added)
69	Bushbuckridge (added)
70	Mayibuye (added)
71	Arthurston (added)
72	Langa (added)
73	Komartport (added)
74	Biyisonto (added)
75	Daveyton (added)
76	Orhovelani (added)
77	Demlani (added)
78	Mbhumba (added)
79	Nyamande (added)
80	Nelspruit (added)
81	Violet Bank (added)
82	Mashonamini (added)
83	Diks (added)
84	Malelana (added)
85	Islington (added)
86	Casteel (added)
87	Zola (added)
88	Polokwane (added)
90	Other village
92	Atholl (added)
.a	Refused to Answer
.b	Skipped
.c	Not asked

High School Codes	
1	Bondzeni High
2	Bunny Khosa High
3	Dumphries High
4	Hlomani High
5	Hobo High
6	Hoyo Hoyo High
7	Ian Mackenzie
8	James Khosa High
9	Luka High
10	Mabarhule High
11	Machaye High
12	Madiba High
13	Madlala High
14	Mandondo High
15	Mawewe High
16	Mchaka High
17	Mzila High
18	Ngaleni Secondary
19	Phulani High
20	Xanthia High
21	Ximoyi High
22	Other, not asked to specify
23	Nyamande (added)
24	Langa (added)
25	Orhovelani High (added)
26	Eric Nxumalo High (added)
27	Mafemani (added)
28	Mkateko High School (added)
29	Khayimani Science and Technology Combined School (added)
30	Witbank High School (added)
31	Makhosana (added)
32	Khanimanba (added)
33	Hloniphho High (added)
34	Technikon College (added)
35	Golden College (added)
36	Lawfeld (added)
37	Frank Maginyana High School (added)
38	Sam Nzima (added)
39	Thulani (added)
40	France High School (added)
41	At university (added)
42	Johannesburg, school not specified (added)
43	Rolle, school not specified (added)
44	Godide Secondary School (added)
45	Wem Private Combined School (added)
46	Hoerskool Sybrand Van Niekerk (added)
47	Sysell Bee Christian School (added)
48	Merry Pebble School (added)
49	Manukusa (added)
50	Freddy Sithole High (added)
51	Jubilee High School (added)
52	Masana High (added)
53	Metropolitan (added)
54	Mogorhoshi (added)
55	Hazyview Private School (added)
56	Magigwana Secondary (added)
57	Acorns to Oaks Secondary (added)
58	Manyangana Secondary (added)
59	Amos Maphanga Secondary School(added)
60	Chayaza Secondary School (added)
61	Ehlanzeni College (added)
62	Lesiba Secondary School (added)
63	M.L. Nkuna (added)
64	Dayimani High School (added)
65	Mugena High School (added)
90	Other
.a	Refused to answer
.b	Skipped
.c	Not asked
.d	Don't know

Primary Schools	
0	Not on this list
1	Matsavana Primary
2	Khayelihle Primary
3	Qetshwayo Primary
4	Nkulungwana Primary
5	Hluvukani Primary
6	Manyakatane Primary
7	Gavazana Primary
8	Mahlahluvana Primary
9	Welani Primary
10	Saringwa Primary
11	Cunningmoore Primary
12	Hokisa Primary
13	Mhlava Khosa Primary
14	Hlanganani Primary
15	Nembe Mhlaba Primary
16	Hundzukani Primary
17	Magudu Primary
18	Tiyani Primary
19	Babati Primary
20	Mketse Primary
21	Njonjela Primary
22	Jan Rikhotso Primary
23	Jongilanga Primary
24	N'wankupane Primary
25	Rhandzekile Primary
26	Hibemandla Primary
27	Mhlahle Primary
28	Vuyelani Primary
29	Dyondzekani Primary
30	JJ Matsane Primary
31	Shalamuka Primary
32	Lisbon Farm School
33	Homuzeya Primary
34	Ndwandwe Primary
35	Mzilikazi Primary
36	Orhovelani Education Center (added)
37	Sam Nzima Private School (added)
38	Bejana Primary School (added)
40	Hokwe Primary School (added)
41	Soniye Primary School (added)
42	Thulamahashe (added)
43	Salani Primary (added)
90	Other (added)
.a	Refused to answer
.b	Skipped
.c	Not asked
.d	Don't know

Education Codes	
1	None
2	Sub-A/Gr 1
3	Sub-B/Gr 2
4	Std 1/Gr 3
5	Std 2/Gr 4
6	Std 3/Gr 5
7	Std 4/Gr 6
8	Std 5/Gr 7
9	Std 6/Gr 8
10	Std 7/Gr 9
11	Std 8/Gr 10
12	Std 9/Gr 11
13	Std 10/Gr 12/Matric
14	Partial Tertiary
15	Completed Tertiary
16	ABET 1
17	ABET 2
18	ABET 3
19	ABET 4
20	Creche
